# RAD21 is the core subunit of the cohesin complex involved in directing genome organization

**DOI:** 10.1186/s13059-023-02982-1

**Published:** 2023-06-28

**Authors:** Yuao Sun, Xin Xu, Wenxue Zhao, Yu Zhang, Keyang Chen, Yongzheng Li, Xiaotian Wang, Mengling Zhang, Boxin Xue, Wanting Yu, Yingping Hou, Chaobin Wang, Wei Xie, Cheng Li, Daochun Kong, Shu Wang, Yujie Sun

**Affiliations:** 1 State Key Laboratory of Membrane Biology, School of Life Sciences, and Biomedical Pioneering Innovation Center (BIOPIC), Peking University, Beijing, 100871 China; 2 Peking-Tsinghua Center for Life Sciences, The National Laboratory of Protein and Plant Gene Research, College of Life Sciences, Peking University, Beijing, China; 3 Center for Bioinformatics, School of Life Sciences, Center for Statistical Science, Peking University, Beijing, 100871 China; 4 Center for Stem Cell Biology and Regenerative Medicine, MOE Key Laboratory of Bioinformatics, School of Life Sciences, THU-PKU Center for Life Science, Tsinghua University, Beijing, 100084 China; 5 Yuanpei College, Peking University, Beijing, 100871 China; 6 College of Chemistry and Molecular Engineering, Peking University, Beijing, 100871 China; 7 Peking-Tsinghua Center for Life Sciences, Academy for Advanced Interdisciplinary Studies, Peking University, Beijing, 100871 China; 8 Breast Center, Peking University People’s Hospital, Beijing, 100044 China; 9 National Biomedical Imaging Center, College of Future Technology, Peking University, Beijing, 100871 China

## Abstract

**Background:**

The ring-shaped cohesin complex is an important factor for the formation of chromatin loops and topologically associating domains (TADs) by loop extrusion. However, the regulation of association between cohesin and chromatin is poorly understood. In this study, we use super-resolution imaging to reveal the unique role of cohesin subunit RAD21 in cohesin loading and chromatin structure regulation.

**Results:**

We directly visualize that up-regulation of RAD21 leads to excessive chromatin loop extrusion into a vermicelli-like morphology with RAD21 clustered into foci and excessively loaded cohesin bow-tying a TAD to form a beads-on-a-string-type pattern. In contrast, up-regulation of the other four cohesin subunits results in even distributions. Mechanistically, we identify that the essential role of RAD21 is attributed to the RAD21-loader interaction, which facilitates the cohesin loading process rather than increasing the abundance of cohesin complex upon up-regulation of RAD21. Furthermore, Hi-C and genomic analysis reveal how RAD21 up-regulation affects genome-wide higher-order chromatin structure. Accumulated contacts are shown at TAD corners while inter-TAD interactions increase after vermicelli formation. Importantly, we find that in breast cancer cells, the expression of RAD21 is aberrantly high with poor patient survival and RAD21 forms beads in the nucleus. Up-regulated RAD21 in HeLa cells leads to compartment switching and up-regulation of cancer-related genes.

**Conclusions:**

Our results provide key insights into the molecular mechanism by which RAD21 facilitates the cohesin loading process and provide an explanation to how cohesin and loader work cooperatively to promote chromatin extrusion, which has important implications in construction of three-dimensional genome organization.

**Supplementary Information:**

The online version contains supplementary material available at 10.1186/s13059-023-02982-1.

## Introduction

In eukaryotes, nuclear chromatin in the interphase is organized in a hierarchical manner. Previous studies using sequencing methods such as Hi-C detected genome-wide interactions, identified the plaid pattern of chromatin [1], and divided chromatin into A and B compartments based on their different eigenvectors. The large compartments are composed of smaller TADs in the 0.2–1.0 Mb range, which are distinguished by the presence of more abundant intra-TAD interactions compared with inter-TAD interactions [2]. High-resolution Hi-C data revealed that chromatin within some TADs is organized into small, stable contact domains anchored by CTCF/cohesin, known as loop domains [3, 4].

The insulator protein CTCF and ring-shaped cohesin complex (also referred as “cohesin” below) were found to be located in the boundaries of TADs and loop domains [2, 3, 5]. In particular, it was demonstrated that CTCF and cohesin are important for the maintenance of loop domains using rapid protein depletion assays based on auxin-inducible degron tagging. These assays found a loss of insulation at most TAD boundaries after CTCF depletion while loop domains rapidly disappeared after cohesin degradation [4, 6–8]. Previous studies have suggested that cohesin-DNA interaction plays an important architectural role in determining chromatin structure during the interphase. Multiple studies, including simulation, in vitro biochemical reconstitution, and single molecule imaging studies, have supported the loop extrusion model to explain the formation of loops and TADs as a process mediated by cohesin progressively extruding DNA until it encounters blocks formed by convergent dimeric CTCF [6, 8–14].

The cohesin complex was originally identified as a tripartite ring playing a role in sister chromatid cohesion in the metaphase, which is constructed by the combination of a V-shaped SMC1/3 heterodimer and the kleisin subunit RAD21 [15, 16]. The association between chromatin and cohesin can be regulated by many factors. For example, cohesin antagonist WAPL can drive the release of cohesin from chromatin [17–19]. Tedeschi et al. depleted WAPL to stabilize the cohesin complex on chromatin and found a vermicelli-like distribution of cohesin by staining, indicating that cohesin plays a role in chromatin condensation [20]. Moreover, high-resolution Hi-C data showed that chromatin loops are enlarged in cells with WAPL deficiency [8, 21], suggesting that the formation of the vermicelli-like distribution is likely caused by excessive chromatin extrusion with the help of over-loaded cohesin. Moreover, PDS5 plays dual roles in cohesin loading [8, 22, 23]. A loading complex called cohesin loader complex NIPBL-MAU2 (also known as SCC2/SCC4, respectively) is required for cohesin loading prior to DNA replication [17]. NIPBL is composed of the head, body and hook domains, and the latter two are sufficient to facilitate cohesin loading [24]. NIPBL was also shown to promote cohesin loading onto circular DNA topologically in vitro by direct interaction at multiple sites on cohesin subunits [25]. After the loading process, the NIPBL is found to hop between different cohesin molecules rapidly in living cells [26]. Using cryo-electron microscopy, a recent study revealed a partial structure of cohesin bound to NIPBL and DNA, which provided the structural basis of cohesin-NIPBL-DNA complex [27]. However, the manner in which cohesin and NIPBL work cooperatively to promote chromatin extrusion is unclear.

Here, we found that up-regulated expression of RAD21, but not other cohesin subunits, leads to a vermicelli-like morphology. To investigate the mechanism of cohesin loading and extrusion in vivo, we used super-resolution imaging to measure the distribution of cohesin subunits. Fluorescence recovery after photo-bleaching and Co-IP results showed that RAD21-loader interaction promotes the formation of a vermicelli-like distribution by facilitating the cohesin loading process. Hi-C data showed that the short distance contact frequency increased in the presence of RAD21 up-regulation, while the long-distance contact frequency decreased. Upon RAD21 up-regulation, chromatin interactions between A and B compartments increased and compartmentalization strength was reduced significantly. Correspondingly, accumulated contacts were shown at TAD corners and inter-TAD interactions increased. These observations support a cohesin loop extrusion model and lead us to propose the unique role of RAD21-loader interaction in cohesin loading on chromatin. Moreover, we combined transcriptome changes and chromatin structure alterations upon RAD21 up-regulation and revealed that the expression level of RAD21 is positively associated with breast cancer.

## Results

### Cohesin is arranged in axial chromosomal domains in cells with up-regulated RAD21

**Fig. 1:**
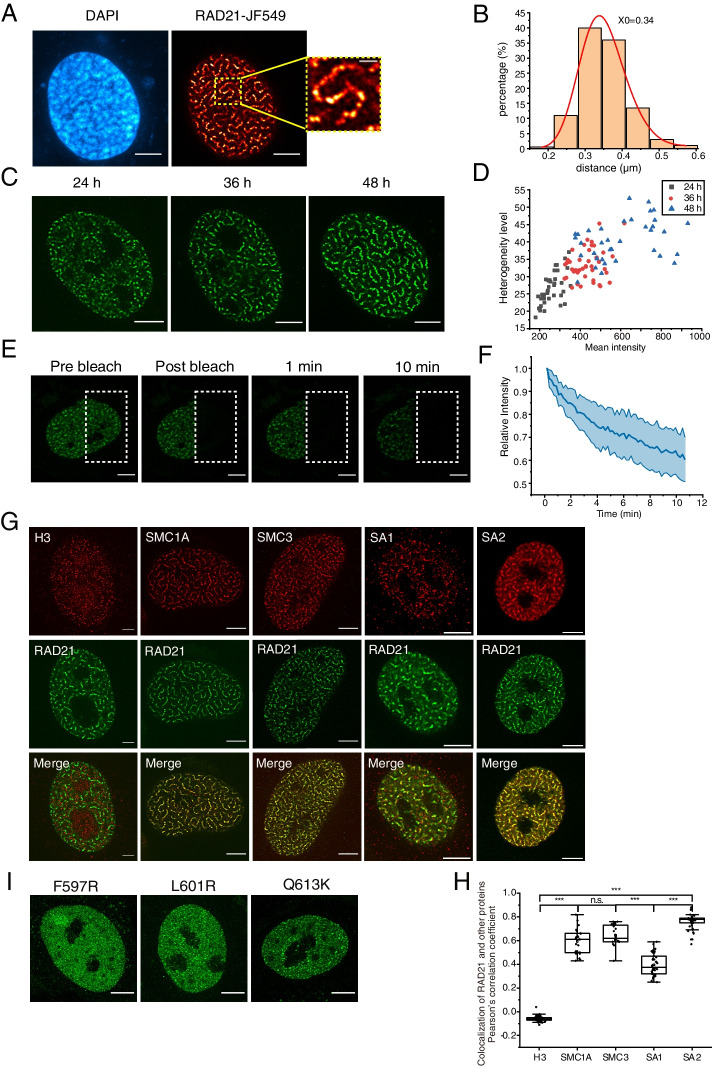
Cohesin is arranged in axial chromosomal domains in cells with up-regulated RAD21. **A** Super-resolution images of HeLa cell transfected with human RAD21. Scale bar, 5 μm. Right, higher magnification of vermicelli. Scale bar, 1 μm. 3 independent experiments. Deconvolution was applied to improve the imaging resolution and signal to noise ratio. **B** Histogram of bead-to-bead distance along the vermicelli fiber in RAD21 over-expressed cell (*n* = 210). **C** Live cell super-resolution images of vermicelli morphology in cells with different after transfected over different time. Scale bar, 5 μm. Deconvolution was applied to improve the imaging resolution and signal to noise ratio. **D** Plots compare mean intensity (x axis) and heterogenetic level (y axis) after RAD21 up-regulation for different time (*n* > 36 per condition). **E** Representative images of an inverse fluorescence recovery after photobleaching (iFRAP) assay after RAD21 up-regulation in HeLa cells. Scale bar, 5 μm. 3 independent experiments. Deconvolution was applied to improve the imaging resolution and signal to noise ratio. **F** The fluorescence signals of unbleached regions were normalized to the first pre-bleach image and plotted (mean ± S.D., *n* = 14 per condition). **G** Super-resolution images of cohesin subunits in the presence of RAD21 over-expression labelled with antibodies. Scale bar, 5 μm. Deconvolution was applied to improve the imaging resolution and signal to noise ratio. **H** Colocalization analysis of vermicelli and protein in (G) (*n *≈ 40 per condition). The lower quartile, median and upper quartile values were labelled in the box. (***P < 0.001, two sample T test). **I** Live cell super-resolution images of three RAD21 mutants in (Additional file 1: Fig. S1I) over-expressed in HeLa cell. Scale bar, 5 μm. 3 independent experiments. Deconvolution was applied to improve the imaging resolution and signal to noise ratio

To investigate the contributions of different cohesin subunits in the cohesin extruding process, we transfected HeLa cells with plasmids containing HaloTag labelled cohesin subunits SMC1A, SMC3, RAD21, SA1 and SA2. The HaloTag-fused cohesin subunits were fluorescently labeled by Janelia Fluor 549 (JF549) in living cells and imaged by a super-resolution spinning disk confocal system (live SR CSU W1 Nikon). We observed that RAD21, as well as chromatin DNA, were both arranged in a vermicelli-like pattern in cells upon RAD21 up-regulation (Fig. 1A, B; Additional file 1: Fig. S1A, B). In contrast, over-expression (OE) of the other four cohesin subunits, SMC1A, SMC3, SA1 and SA2, resulted in even distributions (Additional file 1: Fig. S1C). The super-resolution images of the vermicelli pattern of RAD21 revealed a “beads on a string” pattern (Fig. 1A), which indicates that RAD21 forms clusters on chromatin. Interestingly, the RAD21 “beads” were distributed regularly along chromatin with an average inter-bead distance of 0.34 μm (Fig. 1B). To elucidate the relationship between vermicelli formation and up-regulated RAD21, we observed the distribution pattern of RAD21 in live cells that expressed exogenous RAD21 over different time courses. The data showed that the extent of vermicelli formation (Fig. 1C) was dependent on the expression level of RAD21. We also got the pixel-based intensity distribution among different conditions to calculate their heterogeneity level (described in detail in the Methods section) that represents the accumulation degree of RAD21. The result showed that with increasing time of RAD21 up-regulation, the heterogeneity level was increased with the enhanced fluorescent intensity, revealing that the distribution of RAD21 became more accumulated as its expression level increased (Fig. 1D).

To examine whether RAD21 in the vermicelli are bound to chromatin, we performed inversed fluorescence recovery after photobleaching (iFRAP) experiments, also known as fluorescence loss in photobleaching (FLIP), which can reveal whether RAD21’s diffusion through the nucleus is severely restricted [26, 28]. We observed that the fluorescence intensity of the unbleached region decayed very slowly (Fig. 1E and F). These results suggested that RAD21 binds stably on chromatin in the vermicelli, which could not freely diffuse in the nucleus. To detect cohesin’s residence time on chromatin, we performed the FRAP assay in RAD21-EGFP mES cell line in the absence and presence of RAD21 over-expression for more than 20 min (Additional file 1: Fig. S1D). The results showed that while the instant fluorescence recovery was low in bleached regions after RAD21 up-regulation, cohesin’s residence time on chromatin showed no difference (Additional file 1: Fig. S1E, F). The results indicated that vermicelli could be caused by accumulation of loaded cohesin while cohesin’s unloading kinetics was not affected.

To clarify the integrity of the cohesin ring structure bound to chromatin, SMC1A, SMC3, SA1 and SA2 were immuno-stained in the absence and presence of RAD21 up-regulation. We found that endogenous H3 were distributed homogeneously, while SMC1A, SMC3 and SA2 were colocalized with RAD21 and enriched in vermicelli-like structures after RAD21 up-regulation, which confirmed that the vermicelli-like distribution was caused by the whole cohesin complex rather than RAD21 alone (Additional file 1: Fig. S1G; Fig. 1G, H). In addition, SA1 was less enriched in the vermicelli-like structures in comparison with SMC1A, SMC3 and SA2, which could be due to its lower abundance in the cell. To further dissect the underlying molecular mechanism, we constructed three human RAD21 mutants (F597R, L601R and Q613K) (Additional file 1: Fig. S1H) that are unable to associate stably with SMC1/3 dimers as identified in fission yeast [29]. We found that none of the three RAD21 mutants could change chromatin into vermicelli-like structures (Fig. 1I). Moreover, iFRAP led to a rapid decrease in the fluorescence intensity of the whole cell, indicating that these RAD21 mutants could not bind to chromatin without the formation of a cohesin complex (Additional file 1: Fig. S1I). Taken together, these results also suggest that formation of the vermicelli requires the whole cohesin complex.

### RAD21-loader interaction facilitates vermicelli-like structure formation by promoting cohesin loading

The vermicelli-like phenotype observed upon RAD21 up-regulation could have two explanations. First, RAD21 may act as the limiting factor for cohesin formation so that up-regulation of RAD21 leads to an increased pool of cohesin. Additionally, RAD21 may promote cohesin loading on chromatin and thus bias the loading/unloading balance of cohesin for excessive extrusion of chromatin.

**Fig. 2:**
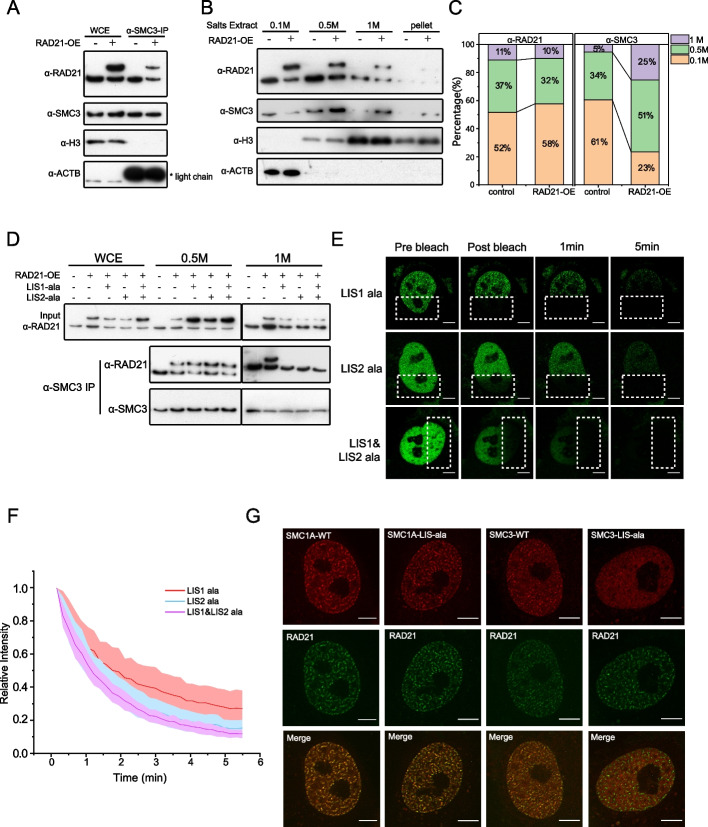
RAD21-loader interaction facilitates vermicelli-like structure formation by promoting cohesin loading. **A** Co-immunoprecipitation and immunoblot assay against SMC3 detect cohesin formation using (whole cell extract)WCE obtained from HeLa cells with endogenous RAD21 or wild-type RAD21-OE. 3 independent experiments. **B** Proteins extracted sequentially at different salt (NaCl) concentrations were immunoblotted with antibodies specific for RAD21, SMC3, H3 or ACTB. 3 independent experiments. **C** The distribution of RAD21 and SMC3 detected in the Western blot shown in A was quantified and normalized to the amount of ACTB and H3. The sample volumes are 1: 0.5: 0.4 (0.1 M: 0.5 M: 1 M). **D** Co-immunoprecipitation and immunoblot assay against SMC3 detect cohesin loading using 0.1 M and 0.5 M salt extract obtained from HeLa cells with endogenous RAD21, wild-type or LIS-Ala-mutant RAD21-OE. 3 independent experiments. **E** Representative images of iFRAP assay of HeLa cells transfected with LIS-Ala-mutant RAD21 (scale bar is 5 μm). 3 independent experiments. **F** The fluorescence signals of unbleached regions were normalized to the first pre bleach image and plotted (mean ± S.D., *n* = 14 per condition). **G** Super-resolution images of cohesin subunits in the absence of RAD21 and wild-type/LIS-Ala SMC1A or SMC3 over-expression (on top of the normal wildtype copies). Scale bar, 5 μm. 3 independent experiments. Deconvolution was applied to improve the imaging resolution and signal to noise ratio

When cohesin complex were isolated from whole cell extract by immunoprecipitation against SMC3 (Additional file 1: Fig. S2A), we found that RAD21 over-expression doesn’t affect SMC3 expression level and doesn’t promote cohesin formation significantly (Fig. 2A). Then we performed salt extraction (Materials and Methods) to quantitatively measure the distribution of RAD21 and SMC3 that are mobile or bound weakly to chromatin (as unloaded cohesin complex or subunit) and that bound stably (as loaded cohesin). The fraction of SMC3 bound to chromatin increased and fraction of unbound SMC3 decreased upon RAD21 up-regulation, indicating accumulation of loaded cohesin complex, while the fractions of bound and unbound RAD21 remain nearly unchanged (Fig. 2B, C).

Moreover, salt extraction and subsequent co-IP experiment showed that with excessive RAD21 the proportion of cohesin was not elevated significantly in the unbound SMC3 pool (Fig. 2D, lane6,7) and the proportion of cohesin was significantly elevated in the chromatin-bound SMC3 pool (Fig. 2D, lane11,12). Considering that the amount of SMC3 with weak chromatin binding decreased (Fig. 2B), we inferred that up-regulated RAD21 reduced the amount of unloaded cohesin. These data collectively suggested that excessive RAD21 promoted cohesin loading on chromatin.

To further support the hypothesis that excessive RAD21 increases RAD21-loader interaction and thus promotes cohesin loading on chromatin, we constructed RAD21 mutants that disrupt interaction with the loader. Previous studies identified the Mis4Scc2–Ssl3Scc4 interaction sites between cohesin subunits in *S. pombe* [25, 30]. To test this hypothesis, HeLa cells were transfected with wild-type RAD21, Loader-Interaction-Site (LIS) deletion RAD21 or LIS-poly-Ala mutations RAD21(Additional file 1: Fig. S2B). LIS-poly-Ala mutations abolished RAD21-NIPBL interaction with no significant effect on cohesin formation (RAD21-SMC3 interaction) and unloading process (RAD21-WAPL interaction) (Additional file 1: Fig. S2C). Salt extraction and subsequent co-IP experiments showed that LIS-Ala mutants accumulated more unbound RAD21 molecules in comparison with wild-type cells and RAD21 over-expressing HeLa cells, and also showed a lack of augmentation of cohesin loading without a significant difference in cohesin formation (Fig. 2D). These results suggested that cohesin loading accumulation is dependent on RAD21-loader interaction, and cohesin with LIS-mutant-RAD21 functions as an intact complex before loading onto chromatin. iFRAP experiments further showed that the vermicelli pattern disappeared when LIS of RAD21 was deleted or replaced by poly-Ala in HeLa cells with RAD21-GFP over-expression (Fig. 2E,Additional file 1: Fig. S2D), while mutant LIS1 showed less modulation compared with LIS2 in iFRAP assays (Fig. 2E, F).

To test whether SMC1A and SMC3-loader interaction dysfunction could disrupt RAD21-mediated cohesin loading, we constructed LIS poly-Ala mutants for SMC1A and SMC3, respectively (Additional file 1: Fig. S2E), and then these two mutants were over-expressed on top of the normal wildtype copies in HeLa cells. In such conditions, the RAD21-vermicelli pattern persisted and colocalized with mutant SMC1A, but not mutant SMC3, which suggested that SMC1A interaction with the loader is dispensable for the cohesin loading process (Fig. 2G).

### Excessively loaded cohesin are anchored by CTCF and bow-tie topologically associating domains

**Fig. 3:**
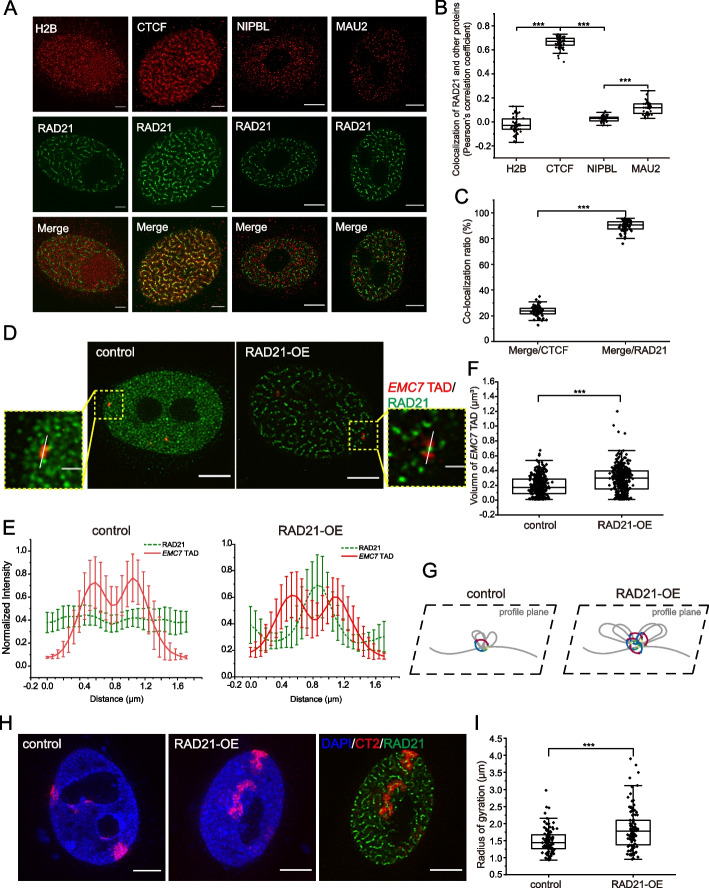
Excessively loaded cohesin are anchored by CTCF and bow-tie topologically associating domains. **A** Super-resolution images of H2B, CTCF, NIPBL, MAU2 in the presence of RAD21 over-expression labelled with antibodies. Scale bar, 5 μm. 3 independent experiments. Deconvolution was applied to improve the imaging resolution and signal to noise ratio. **B** Co-localization analysis of vermicelli and protein in (A) (*n* ≈ 40 per condition). The lower quartile, median and upper quartile values were labelled in the box. (***P < 0.001, two sample T test). **C** Co-localization ratio of CTCF and RAD21 in the presence of RAD21 over-expression (*n* = 54 per condition). (***P < 0.001, two sample T test). **D** Super-resolution images of *EMC7* TAD in the absence or presence of RAD21 over-expression labelled by in situ hybridization with DNA probes, RAD21 was labelled with antibodies. Scale bar, 5 μm. Boxed regions are shown as magnified inserts. Scale bar, 1 µm. 2 independent experiments. Deconvolution was applied to improve the imaging resolution and signal to noise ratio. **E** The normalized intensity profile of boxed region in G measured across the center of the *EMC7* TAD and RAD21 (*n* = 20 per condition). **F** Quantification of volume change of *EMC7* TADs after RAD21 over-expression. (another assay without RAD21 labelled, *n* > 380 per condition). The lower quartile, median and upper quartile values were labelled in the box. (***P < 0.001, two sample T test). **G** Scheme of proposed cohesin-mediated loop model in the absence and presence of RAD up-regulation. **H** Example images of chromosome territory (CT) 2 in RAD21 over-expressed HeLa cell obtained by CT2 probes, JF549 and DAPI. Scale bar, 5 μm. 2 independent experiments. Deconvolution was applied to improve the imaging resolution and signal to noise ratio. **I** Quantification of morphology change of CT2 by calculating the Radius of gyration (*n* = 77 per condition). (***P < 0.001, two sample T test)

Previous studies have suggested that cohesin complexes condense chromatin DNA by progressively extruding DNA with the help of NIPBL and MAU2 [10], meanwhile CTCF works as the boundary to halt the extrusion process resulting in the appearance of TADs. Notably, CTCF was enriched in vermicelli-like structures and colocalized with RAD21, which confirmed the association between CTCF and cohesin when extruding loops met their ends at CTCF sites (Fig. 3A, B). Consistently, a previous study has also reported that CTCF was partially accumulated in vermicelli [20]. The distributions of NIPBL and MAU2 showed no significant difference between wild-type and RAD21-OE cells (Fig. 3A, Additional file 1: Fig. S3A), which was consistent with the dynamic motion of NIPBL during cohesin loading. We also calculated the co-localization ratio of CTCF and RAD21, and we found that CTCF was partially enriched in vermicelli-like structures, while most RAD21 was co-localized with CTCF (Fig. 3C), Which corresponded with the ability of CTCF to function as a boundary element when chromatin was over-extruded by cohesin.

To visualize how TADs are organized in vermicelli-like structures, three TADs (*EMC7* chr15:34007799-35007799, *ACTB* chr7:5260000-5860000, *CD28* chr2:204464723-205264723) were labeled after RAD21 over-expression using fluorescence in situ hybridization (FISH) (Materials and Methods). Interestingly, the RAD21 “beads” were found bow-tying a TAD (Fig. 3D; Additional file 1: Fig. S3B), which was confirmed by normalized intensity profile of the boxed region measured across the center of the *EMC7* TAD and RAD21 (*n* = 20 per condition) (Fig. 3E). We also calculated the volume of *EMC7* TAD and found that the median volume of TAD became larger in the presence of RAD21 up-regulation (Fig. 3F), suggesting that decreased intra-TAD interactions resulted from extrusion that created a trajectory of the chromatin polymer away from the center. These results under different conditions were consistent with the CTCF/cohesin-anchored chromatin loop model [4, 8, 9, 11, 13, 21, 27, 31] (Fig. 3G). Furthermore, we applied 1-h EdU-Cy5 metabolic labeling [32, 33] and found that the area of EdU clusters enlarged significantly after RAD21 was up-regulated (Additional file 1: Fig. S3C, D), which indicated overall enlargement of chromatin domains. To measure the 3D genome organizational change at a larger scale, we performed chromosome painting assays. Chromosome 2 was found to exhibit a fiber-like pattern in the presence of RAD21 over-expression (Fig. 3H, I), indicating that a global conformational effect within a single chromosome was conferred by over-loaded cohesin. We also found that the volume of chromosome 2 became slightly larger after RAD21 over-expression during the formation of vermicelli-like structures (Additional file 1: Fig. S3E). Combining these results, we propose that the structure inside the TAD is more extended (larger volume shown in Fig. 3F) due to the processive extrusion where the chromatin polymer is pulled away from the center.

### RAD21 up-regulation affects genome contacts by stimulating cohesin activity

**Fig. 4:**
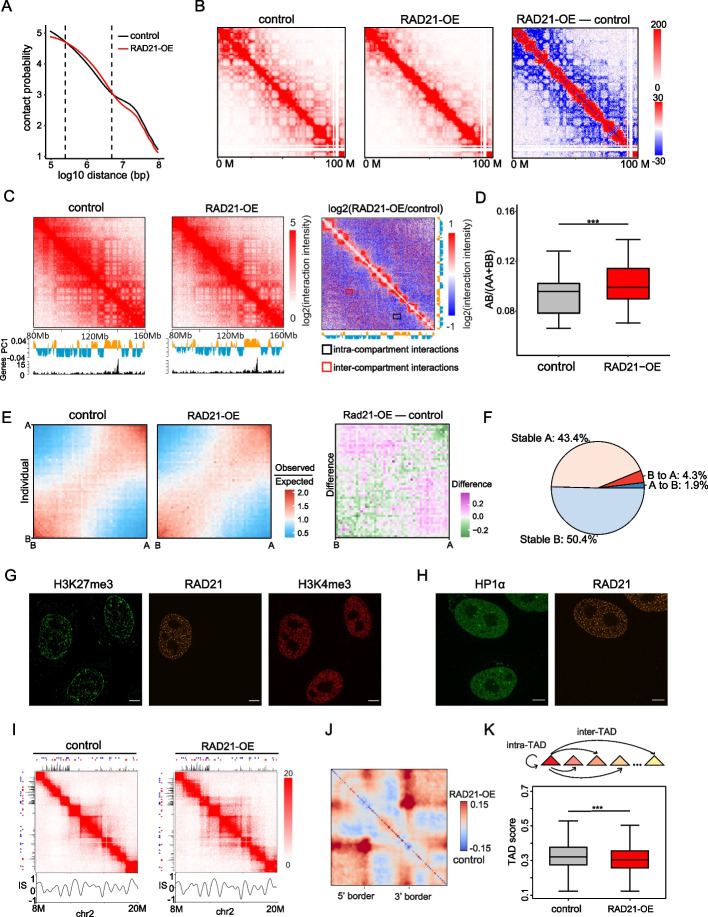
RAD21 up-regulation affects genome contacts by stimulating cohesin activity. **A** Hi-C interaction frequency as a function of logarithmically increasing genomic distance bins for cells with and without RAD21 over-expression. **B** Hi-C contact matrices of chromosome 2 (0–100 Mb) in control and RAD21-OE HeLa cells. Direct minus between RAD21-OE and control matrices is on the right. **C** Normalized Hi-C interaction matrices for chromosome 5 (80–160 Mb) in control and RAD21-OE cells, and differential matrices of genomic regions between control and RAD21-OE cells (resolution: 150 kb). Below the heatmaps are PC1 values and gene density plots. Orange represents compartment A and blue represents compartment B. High gene density regions correlate with compartment A. **D** Ratios of inter-compartment interactions (AB) and intra-compartment interactions (AA + BB) for each chromosome (X chromosome excluded) in control and RAD21-OE cells (***P < 0.001, wilcoxon.test). **E** Average contact frequency enrichment showing the extent of compartmentalization in control and RAD21-OE cells. Direct minus between RAD21-OE and control matrices is on the right. **F** Genome-wide summary of genomic regions switching between A/B compartments in control and RAD21-OE cells. **G** Example immunofluorescence images of control and RAD21 over-expression HeLa cells using anti-H3K27me3 (Green) and H3K4me3 (Red) (scale bar is 5 μm). Transfected HeLa cells are used for the image without FACS sorting RAD21-GFP positive cells. 2 independent experiments. Deconvolution was applied to improve the imaging resolution and signal to noise ratio. **H** Example immunofluorescence images of control and RAD21 over-expression HeLa cells using anti-HP1α (Red) (scale bar is 5 μm). 2 independent experiments. Deconvolution was applied to improve the imaging resolution and signal to noise ratio. **I** Hi-C contact matrices for a zoomed in region on chromosome 2. Matrices are normalized to 160 million contacts, shown resolution is 40 kb. IS, insulation score, shows TAD pattern and insulation score distribution. Above and to the left of the contact matrices the union of CTCF sites identified in wild-type are shown. Red and blue triangles denote forward and reverse CTCF sites, respectively. Black histogram is CTCF ChIP-seq of wild-type HeLa. **J** Aggregate TAD analysis (ATA) calculates the average Hi-C signal across a selected set of TADs. The differential ATA signal between RAD21-OE and control is visualized for all TADs in the size range > 200 kb. Blue indicates a higher signal in the control, red indicates a higher signal in RAD21-OE cells. **K** Contact frequency ratio of intra-TAD and inter-TAD (TAD score) (***P < 0.001, paired t-test)

To study the effect of RAD21 over-expression on genome organization, we performed Hi-C assays in HeLa cells in the presence and absence of RAD21 up-regulation to study the macro-scale consequences on chromosome architecture. The relative contact probability indicated that the genome-wide/global chromosomal contact frequency increased for short distance interactions (0.3–6 Mb) and decreased for long distance interactions (> 6 Mb) in RAD21-OE cells (Fig. 4A). For intra-chromosomal interactions, taking chromosome 2 as an example, Hi-C contact matrices showed that the number of far-cis contacts markedly decreased after RAD21 was up-regulated, whereas the number of short-distance contacts increased (Fig. 4B). The inter-chromosome interaction ratio was significantly decreased after RAD21 up-regulation (Additional file 1: Fig. S4A). These findings are in line with the notion that chromosome territories were more separated from each other during the formation of vermicelli-like structures in RAD21-OE cells.

We next explored the effect of RAD21 over-expression on nuclear compartmentalization, namely compartments A and B, which are defined by the first principal component (PC1) of Hi-C correlation matrices. In RAD21 up-regulated cells, we detected decreased intensity of “checkerboard” patterns, and the distribution of the PC1 scores was less strongly bimodal (Fig. 4C), suggesting a decrease in the compartmentalization level of chromosomes in RAD21-OE cells. To quantify the change of compartmentalization for each chromosome, we calculated the ratio of the interaction frequency between different classes of compartments (AB) versus that between the same classes of compartments (AA and BB) [34]. These ratios were significantly increased in RAD21-OE cells (Fig. 4D), indicating less strict segregation between the A and B compartments. We also calculated the average contact frequency enrichment of compartments ranked by PC1 values, which indicated increasing contact enrichment between different compartment categories and decreasing contact enrichment between similar compartment categories (Fig. 4E). Moreover, 1.9% of genomic regions switched from the A compartment to the B compartment after RAD21 was up-regulated, while 4.3% of genomic regions exhibited the opposite change (Fig. 4F).

We further applied an imaging approach to investigate the effect of RAD21 over-expression on chromatin structure. To visualize the effects of RAD21 over-expression on key epigenomic features associated with genome organization directly, we labeled two histone modifications, histone H3 lysine 4 trimethylation (H3K4me3) and histone H3 lysine 27 trimethylation (H3K27me3), which are associated with active and repressive chromatin in compartments A and B, respectively [1, 3, 35]. RAD21 over-expression had little effect on these features (Fig. 4G). Notably, the distribution of HP1α was unchanged in the RAD21-OE cells, indicating that the formation of vermicelli-like structures was not dependent on heterochromatin (Fig. 4H).

To obtain a better understanding of the increase in the physical volume of TADs after RAD21 was up-regulated (Fig. 3F), we performed TAD calling on whole-genome contact maps and calculated TAD length. Notably, there was no significant change in TAD length after RAD21 was up-regulated (Additional file 1: Fig. S4B). In addition, no significant difference was observed in the number of long (> 500 kb) or short (< 500 kb) TADs (Additional file 1: Fig. S4C), and the insulation scores of TAD borders were similar (Additional file 1: Fig. S4D). These findings indicate that the insulation potential remained unchanged following up-regulation of RAD21. Therefore, over-loaded cohesin can extend TADs when extruding progressively, thereby increasing the volume of TADs, without affecting the length of TADs. In this study, we found that over-expressed RAD21 increased the volume of TADs. One recent imaging study on cohesin depleted cells reported that the contact distances increased significantly for all pairwise interactions both within and between TADs, resulting in TADs expansion [36]. Comparing these results, we proposed that over-loaded cohesin can create a trajectory of the chromatin polymer away from the center when extruding progressively, thereby increasing the volume of TADs. In contrast, the cohesin loss might cause chromatin more disordered and increase the distance within and between TADs.

To further probe how RAD21 over-expression regulates TAD structure, we visualized the interaction heat map at 40 kb resolution. After RAD21 was over-expressed, Hi-C contacts accumulated in TAD corners where CTCF binding sites are located (Fig. 4I). Aggregated TAD analysis (ATA) showed that this effect was global (Fig. 4J). Interestingly, the increase in the signal at the corners of TADs was accompanied by a decreased TAD score (contact frequency ratio between intra-TADs and inter-TADs) (Fig. 4K). Taken together, our results suggest that over-loaded cohesin facilitates the formation of chromatin loops that span the maximal distance within TADs through processive extrusion, which led to increased inter-TAD contact frequency.

### RAD21 up-regulation is involved in breast cancer accompanied with chromatin architecture alternatives

**Fig. 5:**
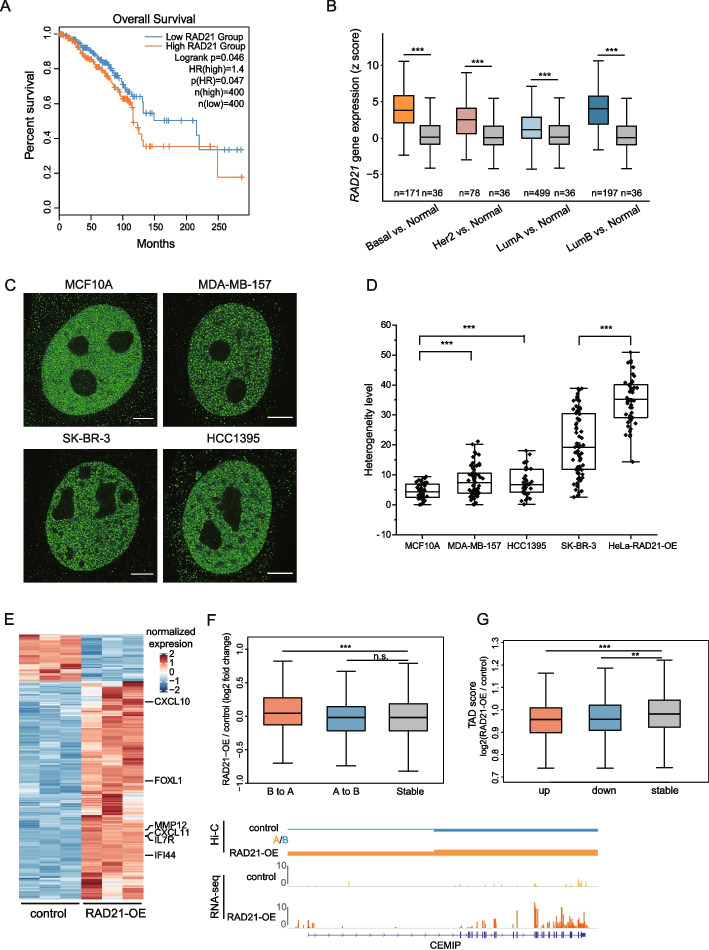
RAD21 up-regulation is involved in breast cancer accompanied with chromatin architecture alternatives. **A** The survival plot based on the expression of RAD21 (orange line denotes the high expression group; blue line denotes the low expression group). **B** Gene expression of RAD21 in breast cancer and normal samples from TCGA database. **C** Example super-resolution images of MCF10A, MDA-MB-157, SK-BR-3 and HCC1395 cells. Scale bar, 5 μm. Deconvolution was applied to improve the imaging resolution and signal to noise ratio. **D** Extent of RAD21 clustering in cells from (**C**) were quantified by heterogenetic level (*n* > 30 per condition). The lower quartile, median and upper quartile values were labelled in the box. **E** Heatmap showing differentially expressed genes (*p* < 0.05, Benjamini-Hochberg; log_2_ Fold change > 1 or log_2_ Fold change < -1) in RAD21-OE cells vs. control cells, RNA-seq analysis was done in HeLa cell line. Some marker genes associated with breast cancer were shown in the right of heatmap. **F** Above: Boxplots showing gene expression changes in regions with compartment switching compared to the stable. *P*-values: Wilcoxon rank-sum test. Bottom: An example of expression level changes of cancer related genes (*CEMIP*) in the regions from B to A compartments upon RAD21 over-expression. **G** Quantification of the difference of TAD score (TAD score = intra-TAD / inter-TAD) between RAD21-OE and control. TADs are stratified into those that contain up-regulated genes or down-regulated genes, or TADs containing genes that show no significant difference in expression. Wilcoxon rank-sum test shows a significant decrease in TAD score between TADs that contain an up-regulated or down-regulated genes and TADs that do not contain a significantly affected genes

Next, we explored the pathological relevance of genome structural disorders caused by RAD21 up-regulation. We first studied clinical data from The Cancer Genome Atlas (TCGA). Through Kaplan-Meier survival analysis, we found that patients with higher RAD21 expression levels did not survive as long as patients with low levels (Fig. 5A). In addition, the RAD21 expression levels in the breast cancer tissues of patients with four cancer subtypes were significantly higher than those measured in normal tissues (Fig. 5B). Moreover, several studies have found that enhanced RAD21 transcription is correlated with increased gene copy number in breast cancer tissue, which is associated with poor prognosis and resistance to chemotherapy [37, 38]. Another study proposed that RAD21 could be a target for breast cancer drugs, because RAD21-targeted siRNA increased the sensitivity of cells to two chemotherapeutic drugs [39]. Therefore, we explored the relationship between genome organization disorders associated with enhanced RAD21 expression and breast cancer oncogenesis and prognosis.

To assess RAD21 aggregation in breast cancer cells with high RAD21 expression levels, we obtained three human breast cancer cell lines with high levels of RAD21 expression [38, 39], and the MCF10A cell line was used as a reference. The RAD21 level of each cell line was measured by western blotting (Additional file 1: Fig. S5A). Super-resolution imaging was used to reveal the endogenous distribution of RAD21. Compared with MCF10A cells, RAD21 was more aggregated and showed a high level of heterogeneity in the three selected breast cancer cell lines, indicating a tendency for the formation of vermicelli-like structures (Fig. 5C, D, Additional file 1: Fig. S5B).

To explore the influence of RAD21 up-regulation, we analyzed gene expression in control and RAD21-OE cells using HeLa cell line. This analysis revealed 649 significantly up-regulated (fold change > 2, *p*-value < 0.05) and 138 down-regulated genes (fold change < 0.5, *p*-value < 0.05) in RAD21-OE cells (Fig. 5E; Additional file 1: Fig. S5C). The top 8 Kyoto Encyclopedia of Genes and Genomes (KEGG) pathways for differentially expressed genes were obtained (Additional file 1: Fig. S5D). Among the top 8 KEGG pathways, cytokine-cytokine receptor interaction, the TNF signaling pathway and the NOD-like receptor signaling pathway are known to play roles in breast cancer [40–42]. In addition, to obtain insight into the relationship between up-regulated RAD21 and breast cancer, we subjected the differentially expressed genes in RAD21-OE cells versus control to gene set enrichment analysis (GSEA). The GSEA identified significant enrichment of mutant TP53, over-expressed KRAS and up-regulated EGFR target genes following RAD21 up-regulation (Additional file 1: Fig. S5E). These gene sets contain several genes that have been widely studied in the context of cancer, including *WNT16*, *CEBPD* and *EGFR*.

Additional analysis was performed to clarify the association between genome structural disorders caused by vermicelli-like structures and differential gene expression. We found that the compartment switch from B to A coincided with higher expression levels, while the switch from compartment A to B was not associated with a significant change (Fig. 5F). One of the up-regulated genes in the compartment B-to-A switch region was cell migration inducing hyaluronidase 1 (*CEMIP*), which has been reported to promote the proliferation and migration of breast cancer cells [43, 44]. A high CEMIP protein expression level is significantly associated with poor patient survival [45]. These results suggest that compartment switching as a result of RAD21 up-regulation can lead to up-regulation of cancer-related genes.

In addition, TAD insulation was also correlated with gene expression. We calculated the TAD score within differentially and stably expressed genes, revealing that both up-regulated and down-regulated genes had reduced TAD score. These results indicated that stronger inter-TAD interactions could affect the expression of genes within the interacting domains (Fig. 5G). These results revealed the connection between breast cancer and genome structural changes upon RAD21 up-regulation.

## Discussion

**Fig. 6:**
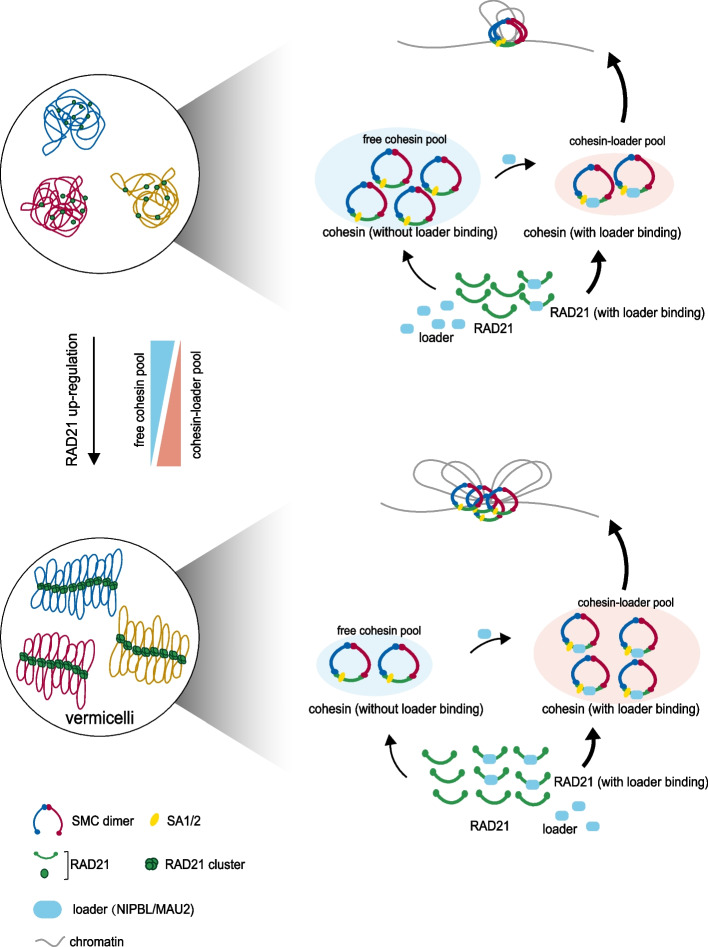
A model illustrating the role of RAD21 as a core subunit of cohesin to extrude DNA and facilitate formation of vermicelli structure. Compared to the normal condition, RAD21 up-regulation increases probability of random collisions between RAD21 and loader, resulting in the assembly of more cohesin-loader complex and thus a decrease in the amount of free cohesin. Then loaded cohesin complex extrudes chromatin progressively, expanding TADs and even chromosome territories

Here, we provide key insights into the molecular mechanism by which RAD21 facilitates the cohesin loading process and thus directing genome organization using live cell super-resolution imaging and biochemical approaches. We observed that excess RAD21 led to intensive chromatin extrusion and vermicelli-like distribution of cohesin. Importantly, we revealed that this process was driven by enhanced loading of the cohesin complex, while the amount of cohesin was not affected significantly. These results suggest that RAD21 acts as a crucial factor in cohesin loading. Mechanistically, RAD21 mutants that are unable to bind the cohesin loader complex cannot lead to the vermicelli-like pattern, indicating that RAD21-loader interaction is an important determinant in the cohesin loading process. Therefore, we propose a model in which up-regulated RAD21 promotes the formation of both RAD21-loader and cohesin, and in turn increases the number of cohesin-loader complexes and cohesin loading on chromatin. Then, the over-loaded cohesin excessively extrudes the chromatin into a vermicelli-like morphology with RAD21 clustered into “beads” and bow-tying a TAD to form a “beads on a string” pattern. (Fig. 6). Hi-C analysis showed that the features caused by RAD21-OE are similar to those after WAPL depletions in genome-wide and compartment scale, but are different at the TAD level. Specifically, the chromosomal contact frequency increased for short distance interactions and decreased for long distance interactions after RAD21-OE. The similar pattern was also found in WAPL-depleted cells reported by Haarhuis et al. As for compartmentalization, over-expression of RAD21 resulted in less strict segregation between A and B compartments. Similarly, the compartments were also less clearly defined in WAPL-depleted cells [46]. On the TAD scale, accumulated contacts were shown at TAD corners and inter-TAD interactions increased after RAD21 up-regulation, which were same as the feature resulted by WAPL depletion [46]. These results suggested that over-expressed RAD21 facilitated the formation of chromatin domains that span the maximal distance within TADs through processive extrusion. Importantly, TAD length and number were found to remain unchanged after RAD21 over-expression while the WAPL-depleted cells displayed greatly reduced TAD number and increased TAD size as reported previously [8]. We inferred that the up-regulated RAD21 may cause TADs to be insulated stably and prevent TADs fusion, resulting in unchanged TAD insulation score, TAD length and number.

According to previous studies, cohesin was found to form a thread-like distribution that has been described as “vermicelli-like”, and chromatin extrusion was shown to be enhanced in mouse embryonic fibroblasts (MEFs) after cohesin was stabilized on chromatin by WAPL depletion. In contrast, our findings showed that over-expression of cohesin subunit RAD21 led to accumulation of cohesin on chromatin and a vermicelli thread-like cohesin distribution in the nucleus of human HeLa cells, while cohesin release from chromatin mediated by WAPL remained unchanged.

Previous studies have established that the loader complex SCC2/SCC4 is essential for cohesin loading onto chromatin [17, 47–49]. In addition, Murayama and Uhlmann showed that RAD21 interacts directly with the loader complex in S. pombe using tiling peptide arrays [25]. In vitro translated Scc1 was found to bind GST (Glutathione S-transferase tag)-Scc2, and the binding region of Scc1 was mapped to residues 126–230 [50]. The different impact of the LIS mutant of each cohesin subunit suggests that RAD21-LIS is crucial for cohesin loading on DNA and further activation. A recent cryo-EM study of the human cohesin-NIPBL-DNA complex shed new light on the structural details of RAD21-loader interactions [27]. The cohesin-NIPBL-DNA complex consists of the V-shaped SMC1-SMC3 heterodimer, the U-shaped NIPBL and the SMC1-SMC3 hinge domains with or without SA1. The complex is stabilized by DNA bound at the central tunnel and surrounding flexible RAD21. The LIS of cohesin is missing from the structure with the exception of LIS2 of RAD21, which mediates RAD21-MAU2 interaction. RAD21 LIS2 is bound by U-shaped SA1 and is located close to the DNA binding interface of SA1, while the missing RAD21 LIS1 is close to the external surface of the NIPBL HEAT repeat (R6, R7, R10), which directly interacts with DNA via its internal surface. Our colocalization results (Fig. 1G, H) indicate that SA2 may be responsible for arresting cohesin in that RAD21 mediated vermicelli pattern.

In addition, RAD21-loader specific interaction may be important in cohesin regulation. For example, recombinant *Chaetomium thermophilum* (Ct) PDS5 competes with Ct Scc2 for binding to Scc1 in vitro in a dose-dependent manner, indicating that it possesses the ability to release Scc2 for the next round of cohesin loading. It was also reported that most (16 of 19) Ct Scc2 mutants were deficient in binding to the N-terminal of Ct Scc1in patients with the human developmental disorder termed Cornelia de Lange syndrome (CdLS) [50]. Considering our findings, it is possible that Scc2 mutants impair cohesin loading and disrupt enhancer-promoter chromatin loop formation at genes with important functions in development, and this result is in line with previous publication that SCC2/SCC4 is engaged in topological DNA entrance into cohesin ring [51].

It is widely accepted that CTCF and cohesin work as boundary elements for chromatin loop extrusion based on the observations that many CTCF binding sites are enriched in the bases of chromatin loop domains in GM12878 cells [3] and the boundary regions of TADs in IMR90 cells [2]. The association of CTCF and cohesin clusters was demonstrated by two-color direct stochastic optical reconstruction microscopy (dSTORM) super-resolution imaging in mES cells, in which these clusters were found to largely overlap [52]. Consistent with this study, our results showed colocalization of CTCF and cohesin in the presence of vermicelli-like patterns. Furthermore, cohesin was found to be essential for the formation and maintenance of TADs and loops. For example, the loop domains disappeared after cohesin was depleted by auxin, and most loop domains recovered in 1 h after auxin was withdrawn in the auxin-inducible degron HCT-116 cell line [4]. Remarkably, Both TADs and loops were greatly reduced after 180 min of auxin treatment in Scc1-mEGFP-AID HeLa cells [8]. Our results showed the distribution of cohesin and TADs directly in the nucleus for the first time. We found that cohesin complexes were enriched in the bases of TADs, which was consistent with the hypothesized model that multiple loop-extrusion cohesin complexes extrude longer chromatin loops constantly within the TAD, the complexes were halted by CTCF binding in a convergent direction at the boundary region of TADs [53]. Longer loops also led to stronger inter-TAD interactions after RAD21 up-regulation.

A previous chromosome spreading study using yeast revealed the presence of 100 or more Scc1 foci [54]. In another study, quantitative western blotting revealed 5–20 cohesin complexes at each cohesin binding site, indicating that cohesin may associate with chromatin in clusters [55]. Similarly, 2–5 molecules in average were found in cohesin clusters in mES cells by live cell STED imaging while CTCF coupled cohesin clusters contain 5–15 molecules [56]. Recently, a new type of phase separation mediated by the SMC protein of the cohesin complex, termed bridging-induced phase separation (BIPS), was reported in a study that showed formation of cohesin-DNA clusters after the application of 10 nM yeast cohesin holocomplexes to double-tethered λDNA. Following the application of cohesin holocomplexes, cluster fusion and rapid recovery after photobleaching were observed, which suggested liquid-like behavior [57]. Consistent with the studies described above, we observed the formation of bead-like clusters in living cells following RAD21 over-expression, and these clusters were located close to TADs labeled by FISH. It is possible that the accumulated cohesin complexes at the boundary regions of TADs mediate the formation and maintenance of chromatin loop domains in the interphase by self-association. However, whether the distribution of cohesin complexes at TAD boundaries is liquid-like cluster or vermicelli-like under physiological conditions is unknown.

Somatic mutations and amplification of RAD21 have been widely reported in both human solid and hematopoietic tumors. RAD21 alterations are relatively common according to TCGA PanCancer atlas studies (7% of all queried patients), which show elevated RAD21 in 20% of ovarian cancer cases and 13% of breast cancer cases. Over-expression of RAD21 has been linked with epithelial breast cancer and has been found to be correlated with poor disease outcome and resistance to chemotherapy [38]. Although the correlation between RAD21 expression and cancer risk is relatively well established, little is known regarding the causes and consequences of RAD21 over-expression in tumorigenesis. In our study, we monitored the outcomes of RAD21 over-expression in HeLa cells and compared the effects of elevated RAD21 on gene expression with TCGA BRCA studies. Unfortunately, we were unable to generate a stable RAD21 over-expression cell line, perhaps due to activation of the TNF-α apoptosis signaling pathway. By comparing transcriptome changes and chromatin structure alterations upon RAD21 up-regulation, we found that chromatin alterations and transcriptional changes following RAD21 up-regulation were consistent. Previous study has shown the importance of cohesin turnover in controlling transcription and propose that a dynamic cycle of cohesin loading and off-loading is critical for gene regulation by mediating promoter and enhancer interactions [58]. Consistently, our model indicating that RAD21 as the core subunit of cohesin may influence cancer-associated gene expression partial due to chromatin excessive extrusion (Fig. 6). Taken together, this study provides important information describing the relationship between chromatin status and the etiology of cancers, which may reveal new targets for clinic treatments.

## Conclusions

Overall, our study provided crucial understanding of how RAD21 supports the cohesin loading process at a molecular level and shed light on the collaborative functioning of cohesin and loader in driving chromatin extrusion, which has significant implications for the development of three-dimensional genome organization.

## Methods

### Cell culture

The human HeLa-S3 immortalized cell line (Catalog No. 30-2004) and human SK-BR-3 cell line (Catalog No. 30-2007) were grown at 37 °C with 5% CO_2_ in high-glucose DMEM (Thermo Scientific, 11965084) containing 10% (v/v) fetal bovine serum (HyClone, SV30087.02) and 100 U/mL Penicillin Streptomycin (Thermo Scientific, 15140163). The human MCF10A cell line (Catalog No. CC-3150) was grown at 37 °C with 5% CO_2_ in MEGM Mammary Epithelial Cell Growth Medium (Lonza, CC-3150) containing supplements required for growth. The human MDA-MB-157 cell line (Catalog No. 30-2008) was grown at 37 ℃ without CO_2_ equilibration in Leibovitz’s L-15 Medium (Thermo Scientific, 11415-064) supplemented with 10% (v/v) fetal bovine serum and 100 U/mL Penicillin Streptomycin. The human HCC1395 cell line was cultured in RPMI 1640 Medium (Thermo Scientific, 11875093) containing 10% (v/v) fetal bovine serum and 100 U/mL Penicillin Streptomycin at 37 °C with 5% CO_2_.

Mouse ESCs were cultured as previous described [59]. Briefly, mESCs were grown on gelatin-coated plates in DMEM supplemented with 10% FBS, penicillin/streptomycin, 5 μM 2-Mercaptoethanol, 2 mM L-glutamine, non-essential amino-acids and 10 ng/mL recombinant Leukaemia-Inhibitory Factor (LIF).

### Live cell imaging and analysis

The Human SMC1A, SMC3, RAD21, and SA1/2 genes were tagged with Halo tag or EGFP and cloned into the pcDNA3.1 vector by Gibson assembly. Unless stated otherwise, cells were plated in glass-bottom dishes, transfected with 2 μg of the indicated plasmids, and allowed to grow for 36 h. Cells were then labelled with 1 μM JF549 (Promega, 6147/5) for more than 15 min and rinsed twice with PBS before imaging. The dishes were then incubated at 37 ℃ with 5% CO_2_ in the incubation chamber of the microscope. Super resolution live-cell imaging was performed using a spinning disk confocal system (Nikon, live SR CSU W) with an EMCCD (iXon DU-897E) mounted on a Nikon Ti-E microscope with a CFI Apo TIRF 100 × Oil (N.A. 1.49) objective.

For iFRAP assays, the cells were transfected with 2 μg of plasmids containing a gene labelled by an EGFP tag for 24–36 h. One pre-bleach image was acquired, after which one half of a nucleus was continuously bleached with the 488 nm laser (100% laser power), and images were acquired by a confocal microscope (Carl ZEISS, LSM880).

Pearson’s correlation coefficient was calculated using ImageJ software with Coloc2 analysis.

For FRAP assays and residence time analysis, we manually counted the fluorescence intensity of the bleached regions at each frame, and carried out photobleaching correction by referring to the fluorescence intensity of the unbleached regions. The photobleaching corrected intensity before and after photobleaching was normalized to 1 and 0. The FRAP curves were fitted by double exponential recovery,

$$F= A\left(1-{e}^{-\frac{t}{{\tau }_{A}}}\right)+B\left(1-{e}^{-\frac{t}{{\tau }_{B}}}\right)$$

Here ${\tau }_{A}$ and ${\tau }_{B}$ are the residence times, and we used the slower one to estimate the residence time. The data fitting in Additional file 1: Fig. S1F was performed as previously reported [52].

### Immunofluorescence

Cells were fixed using 4% (w/v) paraformaldehyde (Electron Microscopy Sciences, 157-8) in PBS (Thermo Scientific, 14190144) for 15 min, followed by three washes in PBS. Permeabilization of cells was performed using 0.5% Triton X-100 (Sigma-Aldrich, T8787-50 ML) in PBS for 10 min, followed by blocking with 5% IgG-free bovine serum albumin (BSA, Jackson, 001-000-162) for 30 min. The indicated primary antibody was added to 5% BSA in PBS at an appropriate dilution and incubated overnight at 4 ℃. After 3 rinses in PBS, cells were incubated with a secondary antibody labeled with an Alexa Fluor dye at a dilution of 1:200 in PBS with 5% BSA for 1 h. The cells were then rinsed with PBS 3 times and fixed using 4% PFA in PBS for 10 min. After 2 rinses in PBS, the cells were finally stored in PBS. Imaging data were acquired using a spinning disk confocal system (Nikon, live SR CSU W1) with an EMCCD (iXon DU-897E) mounted on a Nikon Ti-E microscope with a CFI Apo TIRF 100 × Oil (N.A. 1.49) objective.

For STED (Leica, TCS SP8 STED 3X) imaging, the sample preparation method was identical to the procedure described above, except that the secondary antibody was a donkey anti-Rabbit lgG secondary antibody (Alexa Fluor 594).

**Table:** 

**ANTIBODY**	**IDENTIFIER**	**SOURCE**
Anti-Histone H2B	ab1790	abcam
Anti-Histone H3	ab1791	abcam
Anti-Histone H3 (tri methyl K4)	ab8580	abcam
Anti-Histone H3 (tri methyl K27)	ab6002	abcam
Anti-CTCF	ab128873	abcam
Anti-SMC1A	ab133643	abcam
Anti-SMC3	ab9263	abcam
Anti-SA1	ab4457	abcam
Anti-SA2	ab4463	abcam
Anti-NIPBL	ab220952	abcam
Anti-Scc4	ab183033	abcam

**Table:** 

**ANTIBODY**	**IDENTIFIER**	**SOURCE**
Donkey anti-Rabbit IgG (H + L) Secondary Antibody, Alexa Fluor 488	A-21206	Thermo Scientific
Donkey anti-Mouse IgG (H + L) Highly Cross-Adsorbed Secondary Antibody, Alexa Fluor 488	A-21202	Thermo Scientific
Donkey Anti-Goat IgG (H + L) Highly Cross-Adsorbed Secondary Antibody, Alexa Fluor Plus 488	A32814TR	Thermo Scientific
Donkey anti-Rabbit IgG (H + L) Highly Cross-Adsorbed Secondary Antibody, Alexa Fluor 594	A-21207	Thermo Scientific
Donkey anti-Mouse IgG (H + L) ReadyProbes&trade; Secondary Antibody, Alexa Fluor 594	R37115	Thermo Scientific

### Heterogeneity level analysis

The nucleoli and extranuclear regions were manually selected from the image and the intensity (gray value) of these parts was set to 0. The gray value of all pixels in each image was rescaled to get the same average gray value among different conditions to eliminate the effect of expression level. Considering the radical distance of the vermicelli-like structures was approximately 5 pixels, we used a 5 × 5-pixel box traversing the image and calculated the intensity of the box. If the intensity was 0, we discarded the value, which represented the nucleoli or extranuclear region. To quantify the intensity of different samples, we fitted the distribution of intensity values with a double Gaussian function. The two Gaussian peaks represented the distributions of the sparse regions and dense regions respectively. The heterogeneity level was acquired by calculating the distance between the two peaks. A custom-written MATLAB program was used for the analysis.

### Co-immunoprecipitation (co-IP) assay

GFP-positive HeLa cells transfected with the indicated plasmids were collected using FACS (BeckMan Coulter, Astrios EQ). Generally, 10^6^ cells were sorted, washed twice with PBS, and lysed in a buffer containing 20 mM Tris-HCl (pH 7.5), 100 mM NaCl, 5 mM EDTA, 1% Triton X-100, 0.5% NP-40, 0.1%SDS and protease inhibitors. After incubation at 4 °C for 10 min, the soluble fraction (containing 0.1 M NaCl) was separated from the chromatin fraction by centrifugation at 15,000 × g for 10 min. The chromatin pellet was then incubated in the above buffer with the addition of 0.5 M NaCl at 4 °C for 1 h, followed by centrifugation at 15,000 × *g* for 10 min. The final pellet was resuspended in 1 M NaCl of the above buffer, sonicated for 20 s (1 s on and 1 s off), incubated at 4 °C for 1 h, and then centrifuged. These lysate fractions were subjected to Co-IP with an antibody against SMC3 (abcam, ab9263). Further immunoblot analyses were performed with the following antibodies: H3 (Ruiying Biological Technology, RLM-3038), ACTB (ABclonal, AC026), RAD21 (Thermo Scientific, PA5-54128), and SMC3 (abcam, ab9263).

### Chromosome painting

Cells were plated on a coverslip, transfected with 2 μg of the indicated plasmids, and allowed to grow for 24 h. After fixation in 3:1 (v/v) methanol/acetic-acid, the cells were washed 3 times in PBS. Cell permeabilization was performed using 0.5% Triton X-100 (Sigma-Aldrich, T8787-50 ML) in PBS for 10 min. Next, 10 μL of a commercial probe mixture was applied (Sigma-Aldrich, D-0302-100-FI XCP 2), and the slide was sealed with rubber cement. The cells and probe were denatured by heating the slide on a hotplate at 75 ℃ for 2 min, followed by hybridization in a humidified chamber at 37 ℃ overnight. The slide was then removed from the chamber, and the coverslip was washed in 0.4 × SSC (Sigma-Aldrich, S6639-1L) (pH = 7.0) at 72 ℃ for 2 min. The coverslip was dried, washed in 2 × SSC with 0.05% Tween-20 (Solarbio, T8220) (pH = 7.0) at room temperature for 30 s, rinsed briefly in distilled water, and allowed to air-dry. Next, 10 µL DAPI was applied to a clean slide and covered with the coverslip, followed by sealing with rubber cement. The slide was imaged with a confocal microscope (Nikon, Live SR CSU W1) with a 100 × objective. The imaging data were then processed using Fiji Is Just ImageJ (FIJI).

### Probe design and construction

TADs were identified online (http://3dgenome.fsm.northwestern.edu/chic.php) while probe design and construction were performed with previously described methods [32, 60]. In brief, oligonucleotide probes targeting specific genomic regions were designed following online instructions and the methods of Oligominer (https://github.com/brianbeliveau/Oligominer). The probes were amplified from complex oligonucleotide pools (Hongxun Biotech) by limited cycles of amplification. The templates in the oligonucleotide pools were designed to contain a 32-mer targeting region that is complementary to the genomic sequence, a 30-mer flanking sequence to be hybridized by the secondary probes, as well as two 20-mer primer binding sequences to amplify the probes. The sequences are listed in Additional file 2: Table S1.

For probe construction, we firstly amplified the oligonucleotide pools via 26 cycles of PCR to generate templates for in vitro transcription (Phanta Max Super-Fidelity DNA Polymerase, #P505-d2). The PCR product was column-purified (Zymo DNA Clean and Concentrator, DCC-5) and then converted into RNA via in vitro transcription (HiScribe™ T7 High Yield RNA Synthesis Kit NEB, #E2040S). RNA templates were converted back to DNA via reverse transcription (MAN0012047 TS Maxima H Minus Reverse Transcriptase, Thermo Fisher #EP0751), and the single-stranded DNA products were then purified by alkaline hydrolysis (50 μL of 0.25 M EDTA and 0.5 M NaOH) and column purification (Zymo Research, #D4006). The concentrations of the composites were described in a previous work from our laboratory. The sequences are listed in Additional file 3: Table S2.

### Fluorescence in situ hybridization

For FISH experiments, the cell sample preparation methods were identical to those used for the immunofluorescence experiments as described in the previous study [32]. After fixation using 4% (w/v) paraformaldehyde for 15 min, the samples were incubated in 1 × PBST (1xPBS + 1% (v/v) Triton X-100) for 10 min and then rinsed twice with 1 × PBST. Each sample was then incubated in 100 μg/mL RNaseA (TransGen Biotech, GE101-01) to remove RNA followed by incubation in 0.1 M HCl in 1 × PBST for 10 min, washed 3 times in 1 × PBST, washed 3 times in 2 × SSCT (2 × saline sodium citrate + 1% (v/v) Triton X-100) at RT, and incubated in 2 × SSCT + 50% (v/v) formamide at 4 ℃ overnight. For sample prehybridization, the samples were incubated in 50% formamide (Sigma-Aldrich, 47671) + 2 × SSCT at 78 ℃ for 10 min and then dehydrated by incubation in 70%, 85%, and 100% ice-cold ethanol successively, for 1 min each. For probe prehybridization, synthesized primary probes (5 μL) and secondary probes (1μL, 100 μM) were mixed with 100% formamide (35 μL), after which the probe mixture was incubated in a mixer for 15 min at 37 ℃. Next, pre-warmed 20% (w/v) dextran (Sigma-Aldrich, D8906-10G) (35 μL) was added to the mixture, which was incubated in a mixer for 30 min at 37 ℃. Finally, the mixture was incubated at 86 ℃ for 3 min and cooled on ice immediately.

Samples were then denatured for 3 min at 86 °C and hybridized at 37 °C in a humidified chamber overnight. The hybridized samples were then rinsed twice for 15 min in pre-warmed 2 × SCCT at 60 °C, followed by washing for 10 min in 2 × SSC at room temperature, and stored at 4 °C in 2 × SSC before imaging.

### Cell synchronization and nucleotide labelling

Cells with and without transfection were grown on glass-bottom dishes and synchronized at the G1/S transition by 2 mM thymidine (Sigma-Aldrich, T1895) for 15 h, followed by culturing in fresh DMEM for 10 h, and treatment with 2 μg/mL aphidicolin (abcam, ab142400) for 15 h.

To incorporate EdU in HeLa cells, the cells were bathed with growing medium containing EdU for 30 min after releasing them from the G1/S transition for 1 h, followed by transfection and growth in fresh DMEM for 2 days. The cy5 dye was then conjugated to EdU using the Click-iT EdU Imaging Kit (Thermo Scientific, C10340) according to the manufacturer’s instructions. Imaging data were acquired using the spinning disk confocal system described above.

### Radius of gyration analysis

3D chromosome morphology was first segmented from 3D images using the Imaris Volume module with its default parameters, after which we labeled each 3D chromosome according to its connectivity and obtained the coordinates and intensity (gray value) of each voxel within each chromosome using custom-written MATALB scripts. The radius of gyration (Rg) was obtained as the following formula:

$${R}_{g}=\sqrt{\frac{\sum_{i}{I}_{i}(\overrightarrow{{r}_{i}}-\overrightarrow{{r}_{c}}{)}^{2}}{\sum_{i}{I}_{i}}}$$

where ${\overrightarrow{r}}_{c}$ is the center, ${\overrightarrow{r}}_{i}$ and ${I}_{i}$ are the position and intensity of the $i$-th voxel.

### Cluster size analysis

Clusters were recognized using the Surfaces module including with the Imaris software package. The “diameter of largest sphere” was set to 0.492 μm, the threshold value was set to 25, and the remaining parameters were set to their default values. When the search was complete, we then erased the signal outside the nuclei manually. Finally, the area and other cluster properties were exported by Imaris automatically for the subsequent statistical analysis.

### In situ Hi-C

The in situ Hi-C libraries was generated as previously described [3]. Briefly, cells were grown to approximately 70–80% confluence, washed with PBS, fixed in 1% formaldehyde, and suspended in Hi-C lysis buffer to which 100 U MobI restriction enzyme (NEB, R0147) was added for overnight chromatin digestion. Free ends were labeled with biotin and then ligated together in situ. Crosslinks were reversed, the DNA was sheared to produce 300–500 bp fragments, and then biotinylated ligation junctions were recovered with streptavidin beads. Hi-C libraries were amplified using PCR, constructed according to the NEBnext library preparation protocol (NEB, E7335), and sequenced on the Illumina HiSeq X Ten platform.

### Hi-C data analysis

Hi-C data were processed by HiC-Pro [61]. Briefly, reads were first aligned on the hg19 reference genome. Uniquely mapped reads were normalized using Iterative Correction and Eigenvector (ICE) decomposition and library size. For compartment A/B analysis, HiTC [62] was used to visualize the interaction matrix and calculate the PC1 (at 150-kb resolution). Compartment switches were defined by comparing the PC1 values between RAD21-OE cells and control cells, using zero as the PC1 cutoff. For TAD analysis, ICE-normalized 40-kb resolution matrices were used to detect TAD with a script described by Crane et al. (https://github.com/dekkerlab/crane-nature-2015). Insulation scores were calculated for each 40-kb bin, and the valleys of the insulation score curves were defined as TAD boundaries [63]. CTCF annotation was generated by GENOVA [64]. Aggregate TAD analysis was performed using cooltools (v0.4.0) [65]. The quality control details of Hi-C data can be seen in supplementary file.

### RNA-seq experiments

Total RNA was extracted using the MolPure Cell RNA Kit (YEASEN, 19231ES50). RNA sequencing libraries were constructed using the NEBNext Ultra RNA Library Prep Kit for Illumina® (NEB England BioLabs). RNA-seq paired-end reads were sequenced on the Illumina NovaSeq 6000 platform.

### RNA-seq data analysis

The raw RNA sequences were cleaned using TrimGalore (https://www.bioinformatics.babraham.ac.uk/projects/trim_galore/) and mapped to human reference genome hg19 by STAR (v2.7.1a) with default parameters. All mapped bam files were converted to bigwig using bedtools (v2.24.0) [66]. for visualization in IGV. High-quality mapped reads were quantified using htseq-count (v0.11.3) [67]. Differentially expressed genes were analyzed by DEseq2 [68]. Functional enrichment of previously reported gene sets in the transcriptomes between RAD21-OE and control cells was determined using the GSEA software package [69, 70]. GO enrichment analysis was performed using Enrichr [71].

## Supplementary Information

**Additional file 1: Fig S1.** The Whole Cohesin Complex Is Required for the Formation of the Vermicelli in RAD21-OE Cells. **Fig S2.** Sorting of Artificial RAD21 Cells and Identification of Roles of Cohesin Subunits in Cohesin Loading Process. **Fig S3.** Profile of Vermicelli-like Structures and Chromatin Domain after RAD21 Up-regulation by Super-resolution Image. **Fig S4.** Chromatin Interaction Reorganization and TAD Changes upon RAD21 Up-regulation. **Fig S5.** Aggregated RAD21 in Breast Cancer Cell Lines and Enrichment of Cancer-Related Gene Set upon RAD21 Up-regulation.

**Additional file 2: Table S1.** Oligonucleotide pool sequence of *CD28*, *EMC7* and *ACTB*.

**Additional file 3: Table S2.** Information of probe sequence with Alexa 647.

**Additional file 4.** Review history.

## Data Availability

The microscopy images in figures in additional files as well as the data contributed to the analysis have been deposited in Zenodo (https://doi.org/10.5281/zenodo.7956310) [72]. All Hi-C and RNA-seq datasets have been deposited in GEO under the accession number GSE183186 (https://www.ncbi.nlm.nih.gov/geo/query/acc.cgi?acc=GSE183186). The essential codes of data analysis for reproducible research are available on GitHub (https://github.com/Wenxue-PKU/Publication_RAD21).

## References

[1] Lieberman-Aiden E, van Berkum NL, Williams L, Imakaev M, Ragoczy T, Telling A, Amit I, Lajoie BR, Sabo PJ, Dorschner MO (2009). Comprehensive mapping of long-range interactions reveals folding principles of the human genome. Science.

[2] Dixon JR, Selvaraj S, Yue F, Kim A, Li Y, Shen Y, Hu M, Liu JS, Ren B (2012). Topological domains in mammalian genomes identified by analysis of chromatin interactions. Nature.

[3] Rao SS, Huntley MH, Durand NC, Stamenova EK, Bochkov ID, Robinson JT, Sanborn AL, Machol I, Omer AD, Lander ES (2014). A 3D map of the human genome at kilobase resolution reveals principles of chromatin looping. Cell.

[4] Rao SSP, Huang SC, Glenn St Hilaire B, Engreitz JM, Perez EM, Kieffer-Kwon KR, Sanborn AL, Johnstone SE, Bascom GD, Bochkov ID (2017). Cohesin loss eliminates all loop domains. Cell.

[5] Vietri Rudan M, Hadjur S (2015). Genetic tailors: CTCF and cohesin shape the genome during evolution. Trends Genet.

[6] Gassler J, Brandao HB, Imakaev M, Flyamer IM, Ladstatter S, Bickmore WA, Peters JM, Mirny LA, Tachibana K (2017). A mechanism of cohesin-dependent loop extrusion organizes zygotic genome architecture. EMBO J.

[7] Nora EP, Goloborodko A, Valton AL, Gibcus JH, Uebersohn A, Abdennur N, Dekker J, Mirny LA, Bruneau BG (2017). Targeted degradation of CTCF decouples local insulation of chromosome domains from genomic compartmentalization. Cell.

[8] Wutz G, Varnai C, Nagasaka K, Cisneros DA, Stocsits RR, Tang W, Schoenfelder S, Jessberger G, Muhar M, Hossain MJ (2017). Topologically associating domains and chromatin loops depend on cohesin and are regulated by CTCF, WAPL, and PDS5 proteins. EMBO J.

[9] Alipour E, Marko JF (2012). Self-organization of domain structures by DNA-loop-extruding enzymes. Nucleic Acids Res.

[10] Davidson IF, Bauer B, Goetz D, Tang W, Wutz G, Peters JM (2019). DNA loop extrusion by human cohesin. Science.

[11] Fudenberg G, Imakaev M, Lu C, Goloborodko A, Abdennur N, Mirny LA (2016). Formation of chromosomal domains by loop extrusion. Cell Rep.

[12] Ganji M, Shaltiel IA, Bisht S, Kim E, Kalichava A, Haering CH, Dekker C (2018). Real-time imaging of DNA loop extrusion by condensin. Science.

[13] Kim Y, Shi Z, Zhang H, Finkelstein IJ, Yu H (2019). Human cohesin compacts DNA by loop extrusion. Science.

[14] Nasmyth K (2001). Disseminating the genome: joining, resolving, and separating sister chromatids during mitosis and meiosis. Annu Rev Genet.

[15] Gruber S, Haering CH, Nasmyth K (2003). Chromosomal cohesin forms a ring. Cell.

[16] Haering CH, Lowe J, Hochwagen A, Nasmyth K (2002). Molecular architecture of SMC proteins and the yeast cohesin complex. Mol Cell.

[17] Ciosk R, Shirayama M, Shevchenko A, Tanaka T, Toth A, Shevchenko A, Nasmyth K (2000). Cohesin’s binding to chromosomes depends on a separate complex consisting of Scc2 and Scc4 proteins. Mol Cell.

[18] Gandhi R, Gillespie PJ, Hirano T (2006). Human Wapl is a cohesin-binding protein that promotes sister-chromatid resolution in mitotic prophase. Curr Biol.

[19] Kueng S, Hegemann B, Peters BH, Lipp JJ, Schleiffer A, Mechtler K, Peters JM (2006). Wapl controls the dynamic association of cohesin with chromatin. Cell.

[20] Tedeschi A, Wutz G, Huet S, Jaritz M, Wuensche A, Schirghuber E, Davidson IF, Tang W, Cisneros DA, Bhaskara V (2013). Wapl is an essential regulator of chromatin structure and chromosome segregation. Nature.

[21] Haarhuis JHI, van der Weide RH, Blomen VA, Yanez-Cuna JO, Amendola M, van Ruiten MS, Krijger PHL, Teunissen H, Medema RH, van Steensel B (2017). The cohesin release factor WAPL restricts chromatin loop extension. Cell.

[22] Ouyang Z, Zheng G, Tomchick DR, Luo X, Yu H (2016). Structural basis and IP6 requirement for Pds5-dependent cohesin dynamics. Mol Cell.

[23] Panizza S, Tanaka T, Hochwagen A, Eisenhaber F, Nasmyth K (2000). Pds5 cooperates with cohesin in maintaining sister chromatid cohesion. Curr Biol.

[24] Chao WC, Murayama Y, Munoz S, Costa A, Uhlmann F, Singleton MR (2015). Structural studies reveal the functional modularity of the Scc2-Scc4 cohesin loader. Cell Rep.

[25] Murayama Y, Uhlmann F (2014). Biochemical reconstitution of topological DNA binding by the cohesin ring. Nature.

[26] Rhodes J, Mazza D, Nasmyth K, Uphoff S (2017). Scc2/Nipbl hops between chromosomal cohesin rings after loading. Elife.

[27] Shi Z, Gao H, Bai XC, Yu H (2020). Cryo-EM structure of the human cohesin-NIPBL-DNA complex. Science.

[28] Gerlich D, Koch B, Dupeux F, Peters JM, Ellenberg J (2006). Live-cell imaging reveals a stable cohesin-chromatin interaction after but not before DNA replication. Curr Biol.

[29] Haering CH, Schoffnegger D, Nishino T, Helmhart W, Nasmyth K, Lowe J (2004). Structure and stability of cohesin’s Smc1-kleisin interaction. Mol Cell.

[30] Higashi TL, Eickhoff P, Sousa JS, Locke J, Nans A, Flynn HR, Snijders AP, Papageorgiou G, O’Reilly N, Chen ZA (2020). A structure-based mechanism for DNA entry into the cohesin ring. Mol Cell.

[31] Schwarzer W, Abdennur N, Goloborodko A, Pekowska A, Fudenberg G, Loe-Mie Y, Fonseca NA, Huber W, Haering CH, Mirny L (2017). Two independent modes of chromatin organization revealed by cohesin removal. Nature.

[32] Li Y, Xue B, Zhang M, Zhang L, Hou Y, Qin Y, Long H, Su QP, Wang Y, Guan X (2021). Transcription-coupled structural dynamics of topologically associating domains regulate replication origin efficiency. Genome Biol.

[33] Su QP, Zhao ZW, Meng L, Ding M, Zhang W, Li Y, Liu M, Li R, Gao YQ, Xie XS (2020). Superresolution imaging reveals spatiotemporal propagation of human replication foci mediated by CTCF-organized chromatin structures. Proc Natl Acad Sci U S A.

[34] Du Z, Zheng H, Huang B, Ma R, Wu J, Zhang X, He J, Xiang Y, Wang Q, Li Y (2017). Allelic reprogramming of 3D chromatin architecture during early mammalian development. Nature.

[35] Zhang J, Bellani MA, James RC, Pokharel D, Zhang Y, Reynolds JJ, McNee GS, Jackson AP, Stewart GS, Seidman MM (2020). DONSON and FANCM associate with different replisomes distinguished by replication timing and chromatin domain. Nat Commun.

[36] Hafner A, Park M, Berger SE, Murphy SE, Nora EP, Boettiger AN (2023). Loop stacking organizes genome folding from TADs to chromosomes. Mol Cell.

[37] Porkka KP, Tammela TL, Vessella RL, Visakorpi T (2004). RAD21 and KIAA0196 at 8q24 are amplified and overexpressed in prostate cancer. Genes Chromosomes Cancer.

[38] Xu H, Yan M, Patra J, Natrajan R, Yan Y, Swagemakers S, Tomaszewski JM, Verschoor S, Millar EK, van der Spek P (2011). Enhanced RAD21 cohesin expression confers poor prognosis and resistance to chemotherapy in high grade luminal, basal and HER2 breast cancers. Breast Cancer Res.

[39] Atienza JM, Roth RB, Rosette C, Smylie KJ, Kammerer S, Rehbock J, Ekblom J, Denissenko MF (2005). Suppression of RAD21 gene expression decreases cell growth and enhances cytotoxicity of etoposide and bleomycin in human breast cancer cells. Mol Cancer Ther.

[40] Cruceriu D, Baldasici O, Balacescu O, Berindan-Neagoe I (2020). The dual role of tumor necrosis factor-alpha (TNF-alpha) in breast cancer: molecular insights and therapeutic approaches. Cell Oncol.

[41] Sonnessa M, Cioffi A, Brunetti O, Silvestris N, Zito FA, Saponaro C, Mangia A (2020). NLRP3 inflammasome from bench to bedside: new perspectives for triple negative breast cancer. Front Oncol.

[42] Wang LM, Li J, Liu EZ, Kinnebrew G, Zhang XL, Stover D, Huo Y, Zeng Z, Jiang WL, Cheng LJ (2019). Identification of alternatively-activated pathways between primary breast cancer and liver metastatic cancer using microarray data. Genes-Basel.

[43] Banach A, Jiang YP, Roth E, Kuscu C, Cao J, Lin RZ (2019). CEMIP upregulates BiP to promote breast cancer cell survival in hypoxia. Oncotarget.

[44] Chen Y, Li LH, Zhang JF (2021). Cell migration inducing hyaluronidase 1 (CEMIP) activates STAT3 pathway to facilitate cell proliferation and migration in breast cancer. J Recept Signal Transduct Res.

[45] Xu J, Liu Y, Wang XD, Huang JF, Zhu HJ, Hu ZQ, Wang DF (2015). Association between KIAA1199 overexpression and tumor invasion, TNM stage, and poor prognosis in colorectal cancer. Int J Clin Exp Patho.

[46] Haarhuis JHI, van der Weide RH, Blomen VA, Yanez-Cuna JO, Amendola M, van Ruiten MS, Krijger PHL, Teunissen H, Medema RH, van Steensel B (2017). The cohesin release factor WAPL restricts chromatin loop extension. Cell.

[47] Gillespie PJ, Hirano T (2004). Scc2 couples replication licensing to sister chromatid cohesion in Xenopus egg extracts. Curr Biol.

[48] Lengronne A, Katou Y, Mori S, Yokobayashi S, Kelly GP, Itoh T, Watanabe Y, Shirahige K, Uhlmann F (2004). Cohesin relocation from sites of chromosomal loading to places of convergent transcription. Nature.

[49] Watrin E, Schleiffer A, Tanaka K, Eisenhaber F, Nasmyth K, Peters JM (2006). Human Scc4 is required for cohesin binding to chromatin, sister-chromatid cohesion, and mitotic progression. Curr Biol.

[50] Kikuchi S, Borek DM, Otwinowski Z, Tomchick DR, Yu H (2016). Crystal structure of the cohesin loader Scc2 and insight into cohesinopathy. Proc Natl Acad Sci U S A.

[51] Higashi TL, Uhlmann F (2022). SMC complexes: lifting the lid on loop extrusion. Curr Opin Cell Biol.

[52] Hansen AS, Pustova I, Cattoglio C, Tjian R, Darzacq X (2017). CTCF and cohesin regulate chromatin loop stability with distinct dynamics. Elife.

[53] Bonev B, Cavalli G (2016). Organization and function of the 3D genome. Nat Rev Genet.

[54] Michaelis C, Ciosk R, Nasmyth K (1997). Cohesins: chromosomal proteins that prevent premature separation of sister chromatids. Cell.

[55] Weitzer S, Lehane C, Uhlmann F (2003). A model for ATP hydrolysis-dependent binding of cohesin to DNA. Curr Biol.

[56] Gu B, Comerci CJ, McCarthy DG, Saurabh S, Moerner WE, Wysocka J (2020). Opposing effects of cohesin and transcription on CTCF organization revealed by super-resolution imaging. Mol Cell.

[57] Ryu JK, Bouchoux C, Liu HW, Kim E, Minamino M, de Groot R, Katan AJ, Bonato A, Marenduzzo D, Michieletto D (2021). Bridging-induced phase separation induced by cohesin SMC protein complexes. Sci Adv.

[58] Liu NQ, Maresca M, van den Brand T, Braccioli L, Schijns M, Teunissen H, Bruneau BG, Nora EP, de Wit E (2021). WAPL maintains a cohesin loading cycle to preserve cell-type-specific distal gene regulation. Nat Genet.

[59] Rhodes JDP, Feldmann A, Hernandez-Rodriguez B, Diaz N, Brown JM, Fursova NA, Blackledge NP, Prathapan P, Dobrinic P, Huseyin MK (2020). Cohesin disrupts polycomb-dependent chromosome interactions in embryonic stem cells. Cell Rep.

[60] Boettiger AN, Bintu B, Moffitt JR, Wang S, Beliveau BJ, Fudenberg G, Imakaev M, Mirny LA, Wu CT, Zhuang X (2016). Super-resolution imaging reveals distinct chromatin folding for different epigenetic states. Nature.

[61] Servant N, Varoquaux N, Lajoie BR, Viara E, Chen CJ, Vert JP, Heard E, Dekker J, Barillot E (2015). HiC-Pro: an optimized and flexible pipeline for Hi-C data processing. Genome Biol.

[62] Servant N, Lajoie BR, Nora EP, Giorgetti L, Chen CJ, Heard E, Dekker J, Barillot E (2012). HiTC: exploration of high-throughput ‘C’ experiments. Bioinformatics.

[63] Crane E, Bian Q, McCord RP, Lajoie BR, Wheeler BS, Ralston EJ, Uzawa S, Dekker J, Meyer BJ (2015). Condensin-driven remodelling of X chromosome topology during dosage compensation. Nature.

[64] van der Weide RH, van den Brand T, Haarhuis JHI, Teunissen H, Rowland BD, de Wit E (2021). Hi-C analyses with GENOVA: a case study with cohesin variants. NAR Genom Bioinform.

[65] Flyamer IM, Illingworth RS, Bickmore WA (2020). Coolpup.py: versatile pile-up analysis of Hi-C data. Bioinformatics.

[66] Quinlan AR (2014). BEDTools: the Swiss-army tool for genome feature analysis. Curr Protoc Bioinformatics.

[67] Anders S, Pyl PT, Huber W (2015). HTSeq–a Python framework to work with high-throughput sequencing data. Bioinformatics.

[68] Love MI, Huber W, Anders S (2014). Moderated estimation of fold change and dispersion for RNA-seq data with DESeq2. Genome Biol.

[69] Mootha VK, Lindgren CM, Eriksson KF, Subramanian A, Sihag S, Lehar J, Puigserver P, Carlsson E, Ridderstrale M, Laurila E (2003). PGC-1alpha-responsive genes involved in oxidative phosphorylation are coordinately downregulated in human diabetes. Nat Genet.

[70] Subramanian A, Tamayo P, Mootha VK, Mukherjee S, Ebert BL, Gillette MA, Paulovich A, Pomeroy SL, Golub TR, Lander ES (2005). Gene set enrichment analysis: a knowledge-based approach for interpreting genome-wide expression profiles. Proc Natl Acad Sci U S A.

[71] Kuleshov MV, Jones MR, Rouillard AD, Fernandez NF, Duan Q, Wang Z, Koplev S, Jenkins SL, Jagodnik KM, Lachmann A (2016). Enrichr: a comprehensive gene set enrichment analysis web server 2016 update. Nucleic Acids Res.

[72] Sun Y, Xu X, Zhao W, Zhang Y, Chen K, Li Y, Wang X, Zhang M, Xue B, Yu W, Hou Y, Wang C, Xie W, Li C, Kong D, Wang S, Sun Y. RAD21 is the core subunit of the cohesin complex involved in directing genome organization. 2023. 10.5281/zenodo.7956310. 10.1186/s13059-023-02982-1 PMC10303866 37381036

