# Supplementary material for: RAD21 is the core subunit of the cohesin complex involved in directing genome organization
Source: Genome Biol. 2023 Jun 28;24:155. doi: 10.1186/s13059-023-02982-1 (PMC10303866; doi:10.1186/s13059-023-02982-1)
Supplement: Supplementary file 1 — Additional file 1: Fig S1. The Whole Cohesin Complex Is Required for the Formation of the Vermicelli in RAD21-OE Cells. Fig S2. Sorting of Artificial RAD21 Cells and Identification of Roles of Cohesin Subunits in Cohesin Loading Process. Fig S3. Profile of Vermicelli-like Structures and Chromatin Domain after RAD21 Up-regulation by Super-resolution Image. Fig S4. Chromatin Interaction Reorganization and TAD Changes upon RAD21 Up-regulation. Fig S5. Aggregated RAD21 in Breast Cancer Cell Lines and Enrichment of Cancer-Related Gene Set upon RAD21 Up-regulation. [file 13059_2023_2982_MOESM1_ESM.docx]

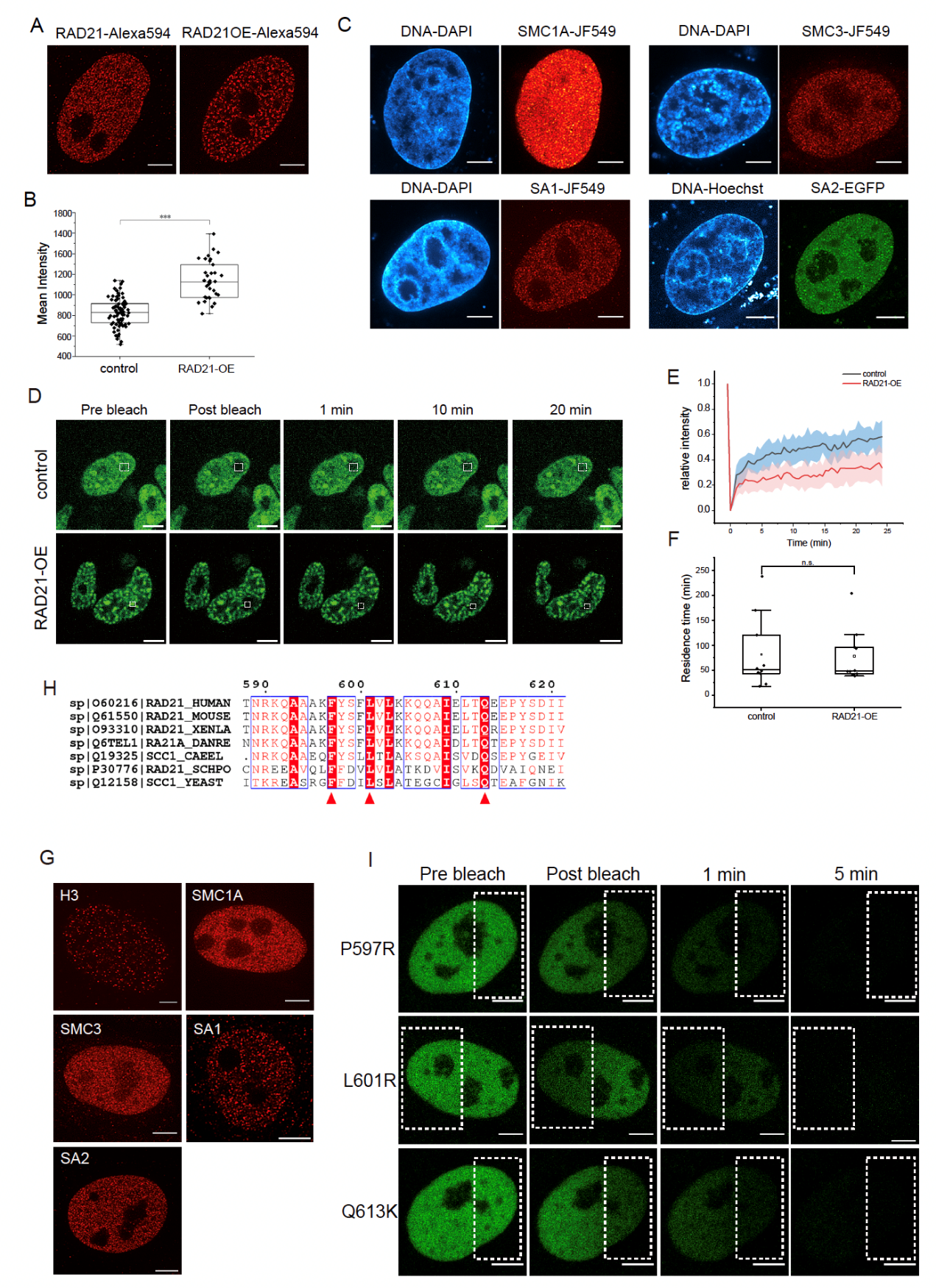


**Fig S1. The Whole Cohesin Complex Is Required for the Formation of the Vermicelli in RAD21-OE Cells**

1. Super-resolution images of endogenous RAD21 and up-regulated RAD21 labeled with antibody. Scale bar, 5 μm. 2 independent experiments. Deconvolution was applied to improve the imaging resolution and signal to noise ratio.
2. ­Measurement of Rad21 expression level by mean intensity quantification (n>35 per condition). (***P < 0.001, paired T test).
3. Super-resolution images of HeLa cell transfected with human SMC1A, SMC3, SA1, SA2. Scale bar, 5 μm. Deconvolution was applied to improve the imaging resolution and signal to noise ratio.
4. Representative images of FRAP assay of SCC1(RAD21)-mAID-GFP ES cells in the absence or presence of RAD21 overexpression (scale bar is 5 μm). 3 independent experiments.
5. The fluorescence signals of unbleached regions were normalized to the first pre bleach image and plotted (mean ± S.D., n = 10 per condition).
6. Residence times for RAD21 in the absence or presence of RAD21 overexpression (n = 10 per condition; two sample T test; n.s., not significant; data fitting was performed).
7. Super-resolution images of endogenous H3 and cohesin subunits labeled with antibody. Scale bar, 5 μm. 2 independent experiments. Deconvolution was applied to improve the imaging resolution and signal to noise ratio.
8. Multiple sequence alignment of partial RAD21 using ClutsterW. Red arrows represent conserved amino acids that are import for RAD21 association with SMC1A/SMC3 dimers.
9. Still images of an iFRAP assay of three RAD21 mutants in Figure 1I over-expressed in HeLa cell. Scale bar, 5 μm.

_
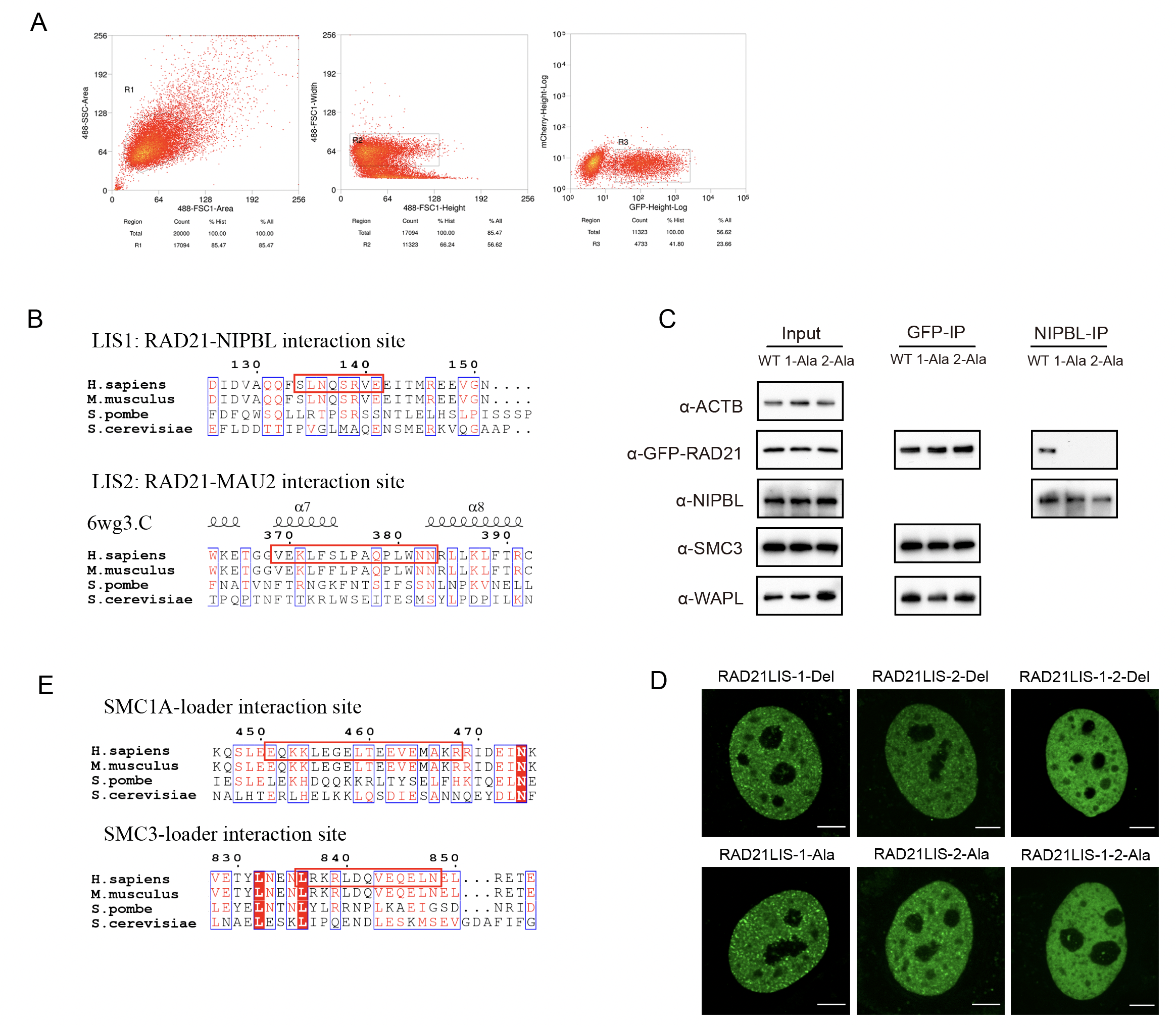
_

**Fig S2. Sorting of Artificial RAD21 Cells and Identification of Roles of Cohesin Subunits in Cohesin Loading Process**

1. The population of RAD21-GFP positive cells were sorted by FACS. 3 independent experiments.
2. Multiple sequence alignment of RAD21 Loader-Interaction-Site between human, mouse, fission yeast and budding yeast using ClutsterW. Red box represents conserved LIS1 and LIS2. Secondary structure elements are generated by structure data of human cohesin (6wg3.C).
3. Co-immunoprecipitation and immunoblot assay against RAD21-GFP or NIPBL detect interaction between SMC3, WAPL, NIPBL with RAD21 using WCE obtained from HeLa cells with wild-type or LIS-Ala-mutant RAD21-OE.
4. Example images of HeLa cells transfected with LIS-deletion or LIS-Ala-mutant RAD21 (scale bar is 5 μm). 3 independent experiments. Deconvolution was applied to improve the imaging resolution and signal to noise ratio.
5. Multiple sequence alignment of SMC1A and SMC3 Loader-Interaction-Site between human, mouse, fission yeast and budding yeast using ClutsterW. Red box represents conserved LIS in SMC1A and SMC3.


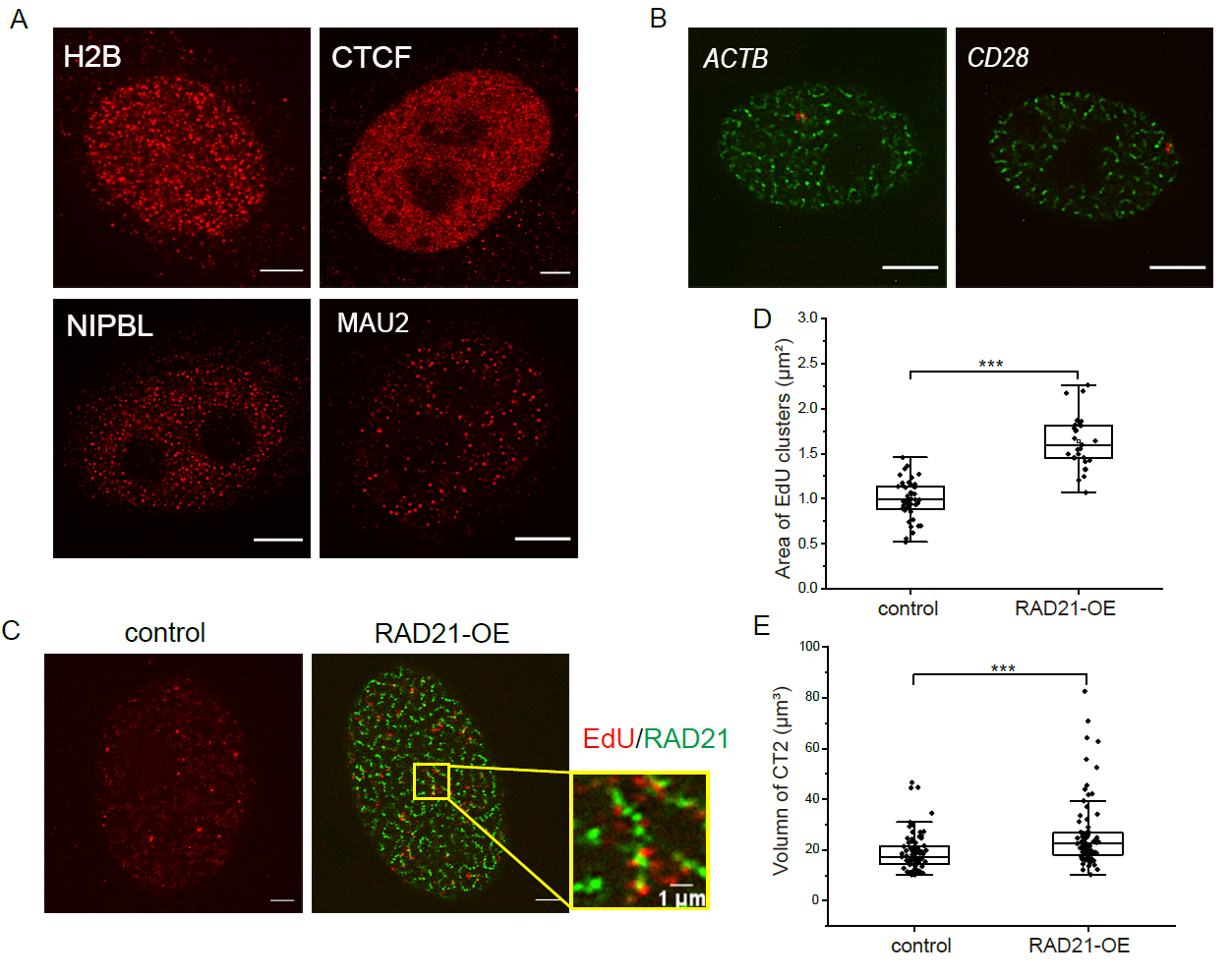


**Fig S3. Profile of Vermicelli-like Structures and Chromatin Domain after RAD21 Up-regulation by Super-resolution Image**

1. Still super-resolution image of endogenous H2B, CTCF, NIPBL and MAU2 labeled with antibody. Scale bar, 5 μm. 2 independent experiments. Deconvolution was applied to improve the imaging resolution and signal to noise ratio.
2. Super-resolution images of *ACTB* and *CD28* TADs after RAD21 over-expression labelled by *in situ* hybridization with DNA probes. Scale bar, 5 μm. Deconvolution was applied to improve the imaging resolution and signal to noise ratio.
3. Super-resolution images of HeLa in the absence or presence of RAD21-OE were labelled with Cy5-EdU. Scale bar, 5 µm. Boxed regions are shown as magnified inserts. Scale bar, 1 µm. 2 independent experiments. Deconvolution was applied to improve the imaging resolution and signal to noise ratio.
4. Quantification of area change of EdU clusters after RAD21 over-expression. (n> 27 per condition). The lower quartile, median and upper quartile values were labelled in the box.
5. Quantification of volume change of CT2 after RAD21 over-expression. (n=77 per condition). The lower quartile, median and upper quartile values were labelled in the box.


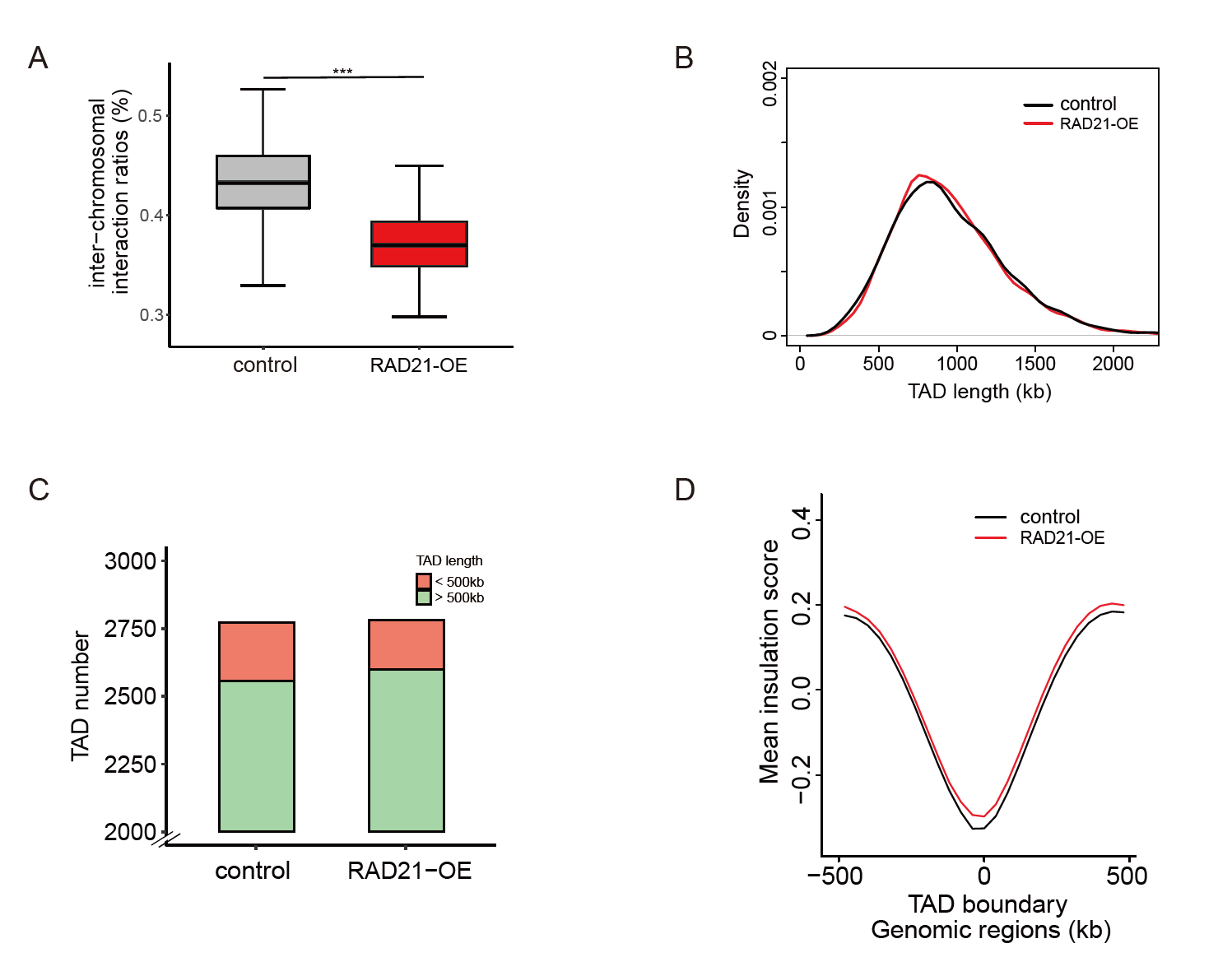


**Fig S4. Chromatin Interaction Reorganization and TAD Changes upon RAD21 Up-regulation**

1. Inter-chromosomal interaction ratios of each chromosome in control and RAD21-OE cells. For each chromosome, inter-interaction ratio is the percentage of inter-interaction in total interaction of this chromosome. (***P < 0.001, Wilcoxon test.)
2. Distribution of TAD length in control and RAD21-OE cells.
3. The number of short (< 500 kb) and long (> 500 kb) TADs.
4. Average insulation score distribution around TAD boundaries (±500 kb) in control and RAD21-OE cells.


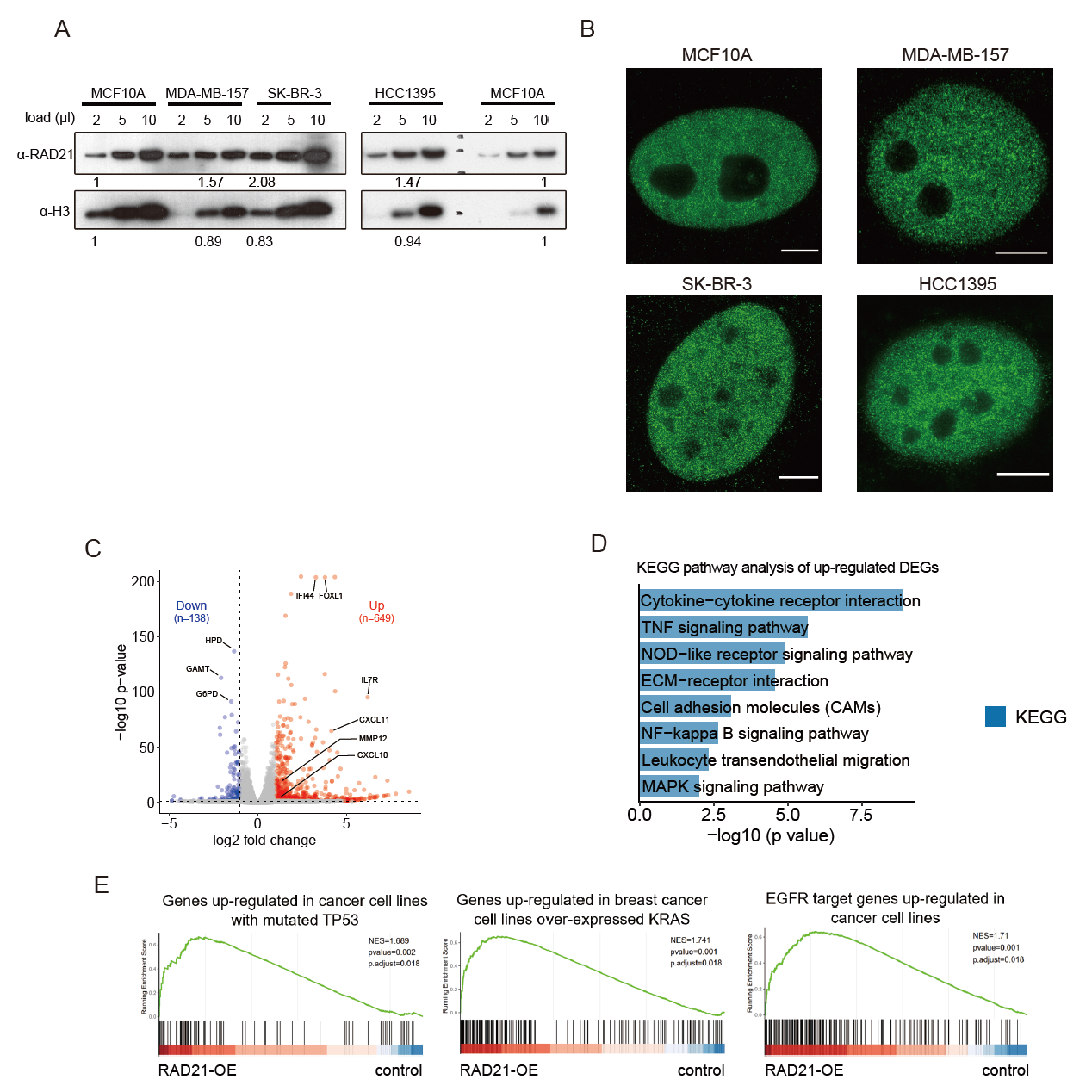


**Fig S5. Aggregated RAD21 in Breast Cancer Cell Lines and Enrichment of Cancer-Related Gene Set upon RAD21 Up-regulation**

1. Immunoblot against endogenous RAD21 and H3 in MCF10A, MDA-MB-157, SK-BR-3 and HCC1395 cells.
2. Example STED images of MCF10A, MDA-MB-157, SK-BR-3 and HCC1395 cells labelled with RAD21 antibodies. Scale bar, 5 μm.
3. Volcano plot showing genes expression level change between control and RAD21-OE HeLa cells. The red and blue dots denote up and down-regulated genes upon RAD21 up-regulation respectively.
4. KEGG pathways enriched (false discovery rate (FDR) < 0.05) in up-regulated genes of RAD21-OE cells. (DEGs, differentially expressed genes)
5. GSEA showing the enrichment of several gene sets associated with basal-like breast cancer (Using oncogenic signature gene sets of the MSigDB).
