# Supplementary material for: RAD21 is the core subunit of the cohesin complex involved in directing genome organization
Source: Genome Biol. 2023 Jun 28;24:155. doi: 10.1186/s13059-023-02982-1 (PMC10303866; doi:10.1186/s13059-023-02982-1)
Supplement: Supplementary file 4 — Additional file 4. Review history. [file 13059_2023_2982_MOESM4_ESM.doc]

**1^st^ round**

**Reviewer 1**

Cohesin complexes are required for the formation of long-range chromatin cis-interactions, which can be detected as chromatin loops and topologically associating domains (TADs) by chromatin conformation capture techniques such as Hi-C (Gassler et al., 2017; Rao et al., 2017; Wutz et al., 2017). Cohesin is thought to generate these chromatin interactions by a process called loop extrusion. In vitro, cohesin possesses such a loop extrusion activity in the presence of a regulatory protein called NIPBL (Davidson et al., 2019; Kim et al., 2019). First evidence for chromatin loop extrusion in cells is just emerging from super-resolution microscopy experiments (Beckwith et al., bioRxiv 2021; Gabriele et al., Science 2022), but the localization of cohesin within chromosome territories has previously been used as a proxy for loop extrusion activity: in immunofluorescence microscopy experiments, cohesin is normally seen distributed evenly on chromatin. However, in the absence of WAPL, a protein that helps to release cohesin from chromatin, cohesin accumulates in axial domains of interphase chromosome territories. These domains, called 'vermicelli', are throught to represent sites at which chromatin loops are anchored by cohesin and at which cohesin accumulates following extended loop extrusion, since WAPL depletion increases the residence time of cohesin on chromatin and thus enables it to extrude longer loops than normally (Tedeschi et al., 2013; Fudenberg et al., 2016). This phenomenon has so far only been observed in cells in which WAPL has been depleted or inactivated by other means.

In this manuscript, Sun et al. report that overexpression of the cohesin subunit RAD21, but not the overexpression of other cohesin subunits, results in the accumulation of cohesin in vermicelli even in the presence of WAPL. Rad21 mutants which are predicted to not interact with NIPBL do not cause this effect upon overexpression. Because NIPBL has previously been proposed to load cohesin complexes onto DNA the authors conclude that RAD21 is required for cohesin loading. The manuscript further reports that RAD21 levels are elevated in certain breast cancer lines, that RAD21 can be observed in vermicelli-like patterns in these cells, and that RAD21 expression levels correlate with poor prognosis in breast cancer patients.

The finding that RAD21 overexpression can result in the accumulation of cohesin in vermicelli is interesting, as are some of the other observations reported in this manuscript. However, I have serious doubts about some of the interpretation of these results. Furthermore, several important experimental controls are missing. Both of these aspects would have to be revised before the manuscript should be considered for publication.

Major points

1. It would be useful to calculate cohesin's chromatin residence times from the iFRAP data shown in Figure 1F. It would also be interesting and important to compare the residence times of cohesin complexes in the absence and presence of RAD21 overexpression. Whereas WAPL depletion leads to vermicelli formation under conditions in which the residence time of cohesin on chromatin is greatly increased, this may be different after RAD21 overexpression, which one would not expect to lead to an increase in cohesin's residence time. If so, the implication would be that vermicelli can be formed simply by increasing the number of cohesin complexes on chromatin without an increase in cohesin's residence time. This would be an interesting finding.

2. Figure 1G and H, the authors find that the cohesin subunits RAD21, SMC1A and SMC3 are enriched in vermicelli, but that SA1 is not, and attribute this difference to the notion that the former three subunits form the cohesin ring, whereas SA1 is peripherally associated with this structure. However, SA1 has been found to be essential for loop extrusion (Davidson et al., 2019), implying that is should also be enriched in vermicelli if the cohesin complexes in these structures contain SA1, unless they contain the SA1 paralog SA2 instead. This possibility has not been considered by the authors. Maybe RAD21 overexpression favors the formation of cohesin complexes containing SA2, which in many cell types has been found to be 3-4 fold more abundant than SA1. It would therefore be interesting to compare SA1 and SA2 staining after RAD21 overexpression. It would also be better to use the standard nomenclature for SA1 and SA2, i.e. STAG1 and STAG2.

3. The authors speculate that RAD21 overexpression facilitates loading of cohesin onto DNA since overexpression of RAD21 mutants that are predicted to not interact with NIPBL does not cause vermicelli formation. However, there are several problems with this interpretation. First, the authors do not show that the RAD21 point mutants generated are unable to interact with NIPBL. This would be important to do. Second, it is not clear if NIPBL functions as a loading factor for extruding cohesin complexes. The original proposal that NIPBL loads cohesin onto DNA was based on the notion that cohesin complexes which mediate sister chromatid cohesion, i.e. trans-contacts between replicated DNA molecules, entrap DNA inside cohesin's ring structure and on the observation that cells lacking NIPBL contain less cohesin on chromatin. The idea was that NIPBL promotes cohesin loading by transiently opening the cohesin ring so that DNA can be entrapped inside this structure to mediate cohesion. However, cohesin can perform loop extrusion, i.e form long-range DNA cis-contacts, without entrapping DNA inside its ring structure, and during loop extrusion NIPBL is continuously required, indicating that its role during this process might not be to load cohesin onto DNA (Davidson et al., 2019). Third, cell lysates contain hetero-dimers of SMC1A and SMC3 (in early papers called '9S cohesin') which do not contain RAD21 (Losada et al., 1998; Sumara et al., 2000; Waizenegger et al., 2000), whereas '14S cohesin' on chromatin has a 1:1:1 stoichiometry of SMC1A, SMC3 and RAD21 (Holzmann et al., 2019). It is therefore well possible that RAD21 overexpression results in the assembly of cohesin complexes from the excess SMC1A-SMC3 hetero-dimers and that the resulting effective increase in cellular cohesin concentration would result in more cohesin complexes on chromatin and more chromatin looping events. The data presented in this manuscript can be equally well - if not better - explained by this alternative interpretation, since cohesin complexes containing overexpressed RAD21 mutants that cannot interact with NIPBL would also not extrude DNA and would therefore not accumulate in vermicelli. The manuscript should therefore discuss and consider both interpretations and needs to amend the conclusion that RAD21 overexpression promotes cohesin loading. RAD21 is presumably required for cohesin loading, but not any more than any of the other cohesin subunits.

Other points (in order in which they appear in the manuscript)

Line 1 and throughout the manuscript, as per standard nomenclature, capital letters should be used for human RAD21.

Line 68, Gassler et al., 2017; Wutz et al., 2017 should be added.

Line 80, Ciosk et al., 2000 did not report findings related to Wapl.

Lines 82, 121/122, the term 'vermicelli' originally only referred to the sites of cohesin staining, not chromatin or chromosome condensation (Tedeschi et al., 2013).

Line 85, Wutz et al., 2017 should be added.

Line 87, 'cohesin' should be 'cohesion'.

Line 90, 'loading complex' should be 'NIPBL', since domains of this protein are being described.

Line 95, 'the structure of cohesin' should be 'a partial structure of cohesin'.

Line 96/97, from the partial cryo-EM structures of cohesin bound to DNA and NIPBL it is not clear whether DNA is located inside cohesin's ring structure. The term 'DNA entrapment' should therefore be avoided in this context.

Line 97, Shi et al., 2020 should be added.

Line 100, the data in this manuscript do not show 'excessive chromatin extrusion' but only a vermicelli-like phenotype.

Line 102 and 203-207, what is the evidence that in the imaging experiments single molecules were detected?

Line 112/113, the claim that 'we … revealed that Rad21 overexpression plays a role in breast cancer by affecting chromatin architecture' is a strong overstatement. The authors did neither present evidence that Rad21 has a role in breast cancer nor that such a role is caused by Rad21 affecting chromatin architecture.

Line 122-124, where the other cohesin subunits overexpressed at levels comparable to those of RAD21?

Line 137, Gerlich et al., 2006 should be cited instead of Rhodes et al., 2017.

Line 211/212, Rhodes et al., 2017 did not provide evidence for DNA extrusion.

Line 214, Figure 3A-C, should mention that Tedeschi et al., 2013 already reported partial accumulation of CTCF in vermicelli.

Line 232/233, Alipour and Marko 2014 did not propose a cohesin/CTCF anchored chromatin loop model

**Reviewer 2**

Review of the manuscript: „Rad21 is the core subunit of the cohesin complex involved in directing genome organization"

In this article, the authors show that overexpression of Rad21 causes chromatin condensation in interphase nuclei and leads to clustering of cohesin in axial structures similar to the so called vermicelli structures. Interestingly, this pattern only occurs if Rad21 is overexpressed but not if the other subunits of the tripartite cohesin ring SMC1 and SMC3 or the associated proteins SA1 or SA2 are overexpressed. Using Western blot and immunoprecipitation experiments, the authors show that overexpression of Rad21 increases the amount of chromatin-bound cohesin whereas the total amount of cohesin is unchanged. Only cohesin containing Rad21 with an intact binding site for NIPBL-MAU2 can be loaded onto chromatin. Besides this, the authors characterize the effect of Rad21 overexpression on the structure of TADs and investigate cancer cell lines overexpressing Rad21.

The work of Sun et al. is of broad interest to others in the field because it addresses the loading of cohesin under physiological conditions. Recent advances are providing impressive molecular insights into the interaction between cohesin and DNA but these studies use reconstituted DNA-protein complexes, for review cf. (Davidson and Peters, 2021; Higashi and Uhlmann, 2022). The data shown by Sun et al. are from a cell system and show a correlation between the amount of RAD21 and the amount of cohesin loaded to chromatin. However, evidence is mainly based on immunoprecipitation and Western blot data and do in contrast to Line 47 - 49 of the manuscript not provide new molecular insights into loading or loop extrusion process.

Major concerns

Line 47 -49 This claim does not seem to be proven by the data in the manuscript

Line 146 The immunostaining of SA1 (Fig. 1G and Fig S1D) does not look convincing, there seems to be a problem with accessibility of the antibody, see e.g. (Schnell et al., 2012)

Scaling / display of fluorescence microscopy images should generally be improved. In some cases the images are so dark that the images cannot be assessed. Examples are: Fig. 1E, G / 2G / 4G, H. Many images look deconvolved, please specify if deconvolution was used.

The authors use iFRAP to investigate whether cohesin is stably bound to chromatin or not. However, the data in Fig. 2 show that different pools of bound and unbound cohesin exist. Moreover, unbound RAD21 and RAD21 bound to NIPBL-MAU2 also exist in parallel (Fig. 6). These pools can be examined using standard FRAP. The experiment would require an analysis of the kinetics of the reappearance of fluorescence after a bleaching event. Of course, a full kinetic analysis of all mutants is beyond the scope of this manuscript, but the conclusions from Fig. 2 and the single molecule data (Figure S2E) could be substantially supported by classical FRAP experiments.

The two histograms in Figure S2E look identical, therefore the respective conclusions (line 205 - 206) cannot be assessed completely. However, from the statement in line 172 - 173 one had to expect a significant pool of RAD21 in cells overexpressing this protein. The authors should clarify this. In the methods section a description of the setup and the experimental conditions of the single molecule experiment are missing. In particular, it is unclear which cells were examined. Possibly only control cells were used. In this case the authors should consider to also measure cells overexpressing Rad21 which should result in an increase of the mobile fraction.

Figures 1H, 3B, 3C, 3F, 3I, S1B indicate significant differences, but the details of statistical tests performed on the data are not specified. The statistics in the other figures are outside of the expertise of the reviewer.

Minor corrections

Line 95 - 97 Please consider inserting citation for (Shi et al., 2020)

Line 110 The importance of the interaction between RAD21 and NIPBL-MAU2 is well established in the literature. The authors should focus on the new point that the Rad21-loader interaction is one mechanism controlling the amount of cohesin which is loaded to chromatin in living cells.

Line 118 The authors should mention that these protein were not endogenously tagged by genome editing but instead overexpressed by using plasmids.

Line 136 For some readers the term "fluorescence loss in photobleaching (FLIP)" might be more common than the term iFRAP. The authors could for example insert something like "also known as fluorescence loss in photobleaching (FLIP)".

Line 143 The manuscript contains many abbreviations, with H3 being a common abbreviation, but others such as GST-Scc2 in line 387 are not. Please introduce abbreviations in a consistent manner maybe by using a list of abbreviations.

Line 203 I do not see how the described single molecule imaging helps to quantify the loading capacity. Please clarify this or delete this statement.

Line 231-232 Sentence structure

Line 258 The term segregation might be confused with the chomatid segregation during mitosis. Therefore it might be helpful to use another term in line 258.

Line 361 - 363 here is written: "Importantly, we revealed that this process was driven by enhanced loading of the cohesin complex, while the amount of free cohesin was unaffected" This would mean that the overall amount of cohesin is increased! However the claim in line 172- 173 is "up-regulation of Rad21 increased the amount of Rad21 by 2.5-fold while the total amount of cohesin showed almost no difference". Please clarify this discrepancy.

Line 378 please insert citation

Line 399 Figure S1D does not contain colocalization information. Therefore the conclusion cannot be verified

Line 407 From published models and loading mechanisms most people from the field would expect that SCC2 mutants impair cohesin loading, cf. Figure 1 in (Higashi and Uhlmann, 2022). Possibly the authors could argue that findings in the manuscript are in line with others.

Line 449 Please add citation.

Line 566 Please provide the oligonucleotide probe sequences in an supplementary table. Furthermore, you could indicate which readout oligonucleotide was used and which dye was conjugated with it.

Line 584 Please cite the previous work

Line 921 Typo in the term CTCF

Line 928 Typo in EMC7

929 EMC7 is set to italic only here

Figure 2E and F are not mentioned in the text.

Supplement

Line 17 - 19 Disrupted title.

Line 64 - 65 The advantage of using STED microscopy is not obvious. The authors should clarify if really STED images are shown, as they look very similar in resolution to the others.

Literature

Davidson, I.F., and Peters, J.M. (2021). Genome folding through loop extrusion by SMC complexes. Nat Rev Mol Cell Biol 22, 445-464. 10.1038/s41580-021-00349-7.

Higashi, T.L., and Uhlmann, F. (2022). SMC complexes: Lifting the lid on loop extrusion. Curr Opin Cell Biol 74, 13-22. 10.1016/j.ceb.2021.12.003.

Schnell, U., Dijk, F., Sjollema, K.A., and Giepmans, B.N. (2012). Immunolabeling artifacts and the need for live-cell imaging. Nat Methods 9, 152-158. 10.1038/nmeth.1855.

Shi, Z., Gao, H., Bai, X.C., and Yu, H. (2020). Cryo-EM structure of the human cohesin-NIPBL-DNA complex. Science 368, 1454-1459. 10.1126/science.abb0981.

**Reviewer 3**

Overall the work is exciting and represents an important contribution to the field. I have a few concerns, mostly with respect to the clarity of presentation in a number of places that, I think the authors may wish to address, which I describe below.

PRIMARY CONCERNS:

1) Rad21 induces vermicelli-like chromosomes overlapping a vermicelli-like Rad21 beads-on-a-string configuration. This result was cool and striking. Three possible explanations are natural: that Rad21 is in limiting concentration, that exogenous Rad21 enhances loading, or that exogenous Rad21 interferes with unloading. The authors do a reasonable job in Figure 2 in arguing against the 1st explanation. From my reading I think it may still be possible that exogenous, tagged Rad21 interferes with unloading of cohesin. In the discussion the authors write: "In contrast, our findings showed that over-expression of cohesin subunit Rad21 led to accumulation of cohesin on chromatin and a vermicelli thread-like cohesin distribution in the nucleus of human HeLa cells, while cohesin release from chromatin mediated by Wapl remained unchanged. We therefore inferred that Rad21 is an im-portant factor that affects cohesin loading independent of Wapl." .., but I don't believe that the au-thors showed any data supporting these conclusions about Wapl.

1.1) If the authors bleach a region of the cell containing a fluorescently tagged Rad21, and measure recovery over time on timescales longer than currently estimated residence time of cohesin (~20 min an estimate probably good to within an order of magnitude), does it recover? Ideally this would be compared to the cohesin endogenously tagged with the same fluor (such lines exist, for example Hansen and colleagues engineered a Halo tagged cohesin in mESC, which could also be used for transfection of the over-expression construct. Making new endogenously tagged lines is non-trivial project, but if the expertise is available in house can be done in a few weeks to a month).

1.2) Does over-expression of an untagged Rad21 also create the vermicelli (in the DAPI channel)? This would rule out if the Halo-tag was impeding the unloading.

2) The differences in Rad21 organization in the cancer lines examined were very interesting. It might be beyond the scope of this work, but it would bolster the findings if by Hi-C these lines exhibited similar trends to enhanced near-cis and decreased far-cis interaction, as well as strengthened chromosome territories and weakened compartments. I'd be inclined to support publication even lacking these additional experiments, but I do think they'd enhance the impact of the paper and strengthen the claims here.

3) It would have been nice to see a comparison or at least a discussion of Rad21 OE with the vermicelli chromosomes observed after WAPL degradation. For example, a more direct comparison of features reported in the literature due to removal of WAPL to the measures in this paper (including HiC fea-tures of TAD and loop sizes the minimum). If it is feasible in reasonable timeframe, experiments knocking down WAPL and comparison of Rad21 localization patterns (by IF or if the authors have and endogenously tagged Rad21 cell line) would be insightful.

MINOR CONCERNS

Introduction

1) "High-resolution Hi-C data revealed that chromatin within some TADs is organized into small, stable contact domains anchored by CTCF/cohesin, known as loop domains (Rao et al., 61 2014; Rao et al., 2017)."

In the understanding of this reviewer, "contact domains" aren't really distinct from TADs in stability, size, or frequency of having corner loops. The Aiden lab preferred "contact domain" instead of "topologically associating domain", perhaps to avoid the suggestion that TADs have something to do with the mathematical idea of topology (knots).

2) "Wapl can drive the release of cohesin from chromatin(Ciosk et al., 2000; Gandhi et al., 2006; Kueng et al., 2006) … cells with Wapl deficiency(Haarhuis et al., 2017), suggesting that the formation of the vermicelli-like distribution is likely caused by excessive chromatin extrusion with the help of over-loaded cohesin. Inversely, PDS5 is necessary for the maintenance of cohesin(Panizza et al., 2000)."

I understood PDS5 to be an unloader like WAPL (e.g. see https://www.ncbi.nlm.nih.gov/pmc/articles/PMC6952610/), though the authors text left me with the opposite impression.

Results

3) the supplemental figures were missing from the documents I received.

4) it would be nice to see in Fig.1 the wild type (not Rad21 OE) chromosomes by DAPI and/or IF for Rad21. (this may already be in the supplement?).3) In Fig 1 and 2, It was unclear why the authors use iFRAP instead of FRAP, I thought the lack of recovery in the bleached region was much more convincing than the gradual photo-bleaching in the unbleached region. It seems to me a straight-forward quantification of the FRAP would be warranted here. I would also recommend a longer time course for the FRAP, given previous estimates that the loading/unloading kinetics of cohesin/Rad21 are much slower than for other transcription factors.

5) "We also got the pixel-based intensity distribution among different conditions to calculate their heterogeneity level (described in detail in the Methods section), which revealed that the distribution of Rad21 became more aggregated as its expression level increased (Figure 1D). "

Fig 1D is unclear in the plot and the main text. Either briefly explain in the text what the reader is supposed to take away from this panel or remove it. But it is currently unclear to me in what way this shows Rad21 being more aggregated. Panel C does make it look like the RAD21 vermecilli become more clearly resolved, but it is unclear from C whether this is just due to the increased levels and the saturation of the image pixels shown at 48hrs.

6) The clarity of the text could be improved for the general reader. Specifically:

- Line 183: the authors could be more specific in writing out 'loader', do they mean NIPBL ?

- Line 191: what do the authors mean by 'cohesin formation'. Does cohesin here refer to SMCs + SA + Rad21 complex?

- the authors define 'LIS' as Rad21-loader-interaction-site in line 187-188. It is a little confusing to then have LIS for SMC1a and SMC3. A sentence and or schematic of the position of the site within the protein in the text to introduce the knowledge about the loader interaction sites for each subunit would be helpful.

-Fig. 2E - labeling Fig. 2E with not just LIS but Rad21-LIS-ala (as compared to 2G which is SMC1A or SMC3). Adding wt Rad21 here as a comparison would be good, since this is GFP tagged and not Halo tagged. Also in 2F, wt as a comparison would be helpful to compare with what is expected.

7) Fig 2G SMC3 mutant -was unclear if this is an over-expression of the SMC3 loading mutant on top of the normal wildtype copies, or an edit of one or both of the endogenous copies. It would help the flow of the main text if the authors clarified in this in the main text directly - I don't think this sort of key information should be restricted to the supplemental methods.

8) Fig. 3A/B: comparison for non-overexpressed Rad21 would be useful for how much CTCF-Rad21 colocalization happens in wt cells.-Fig. 3D: is control = Rad21 wt ?

9) "Bow-tying the TAD" (Fig 3E-G) This was interesting, though the bipartate nature of the structure in control condition as well as the Rad21 OE condition might reflect the cell cycle / paired sister chromatids - especially with the smallish n=20, differences in the fraction of G2 vs. G1 cells in each sample might contribute to difference in both volume and degree of separation (especially if RAD21 OE affects cell cycle / impairs M entry for example). This could be readily tested by adding a cell cycle marker like geminin into the quantification in parallel with the RAD21 label.

10) Does Rad21 OE effect cell cycle? (see also 6)

11) I was unable to see these results due to the missing supplement: "1-hr EdU-Cy5 metabolic labeling(Li et al., 2021; Su et al., 2020) and found that the area of EdU clusters enlarged significantly after Rad21 was up-regulated (Figures S3C and S3D)", how many clusters? What time point afte metabolic labeling? Same cell cycle? Can enlarged EdU clusters be due to enhanced transcription?

12) "median volume of TAD became larger in the presence of Rad21 up-regulation (Figure 3F), suggesting that decreased intra-TAD interactions resulted from excessive extrusion(Liu et al., 2021). These results under different conditions consistent with the CTCF/cohesin-anchored chromatin loop model" and

"inside the TAD is more extended (larger volume shown in Figure 3G) due to the excessive extrusion of chromatin caused by overloaded cohesin, and therefore the interior structure of the chromatin region is more extended." - I suspect this is an artefact of the limiting labeling resolution (that more cohesin actually compacts the chromosome, not expanding it, but I see why the authors conclude the opposite. (Fig 3F, not G, no?) I think it's overstating things to say this expansion is consistent with the loop extrusion model - I think that's basically incorrect. I appreciate the cartoon intuition that extrusion creating a trajectory of the chromatin polymer away from the center - but this is not how it behaves in the chromatin polymer simulations cited nor how a floppy polymer is expected to behave in a crowded nuclear environment.

13) "over-loaded cohesin can extend TADs when extruding progressively, thereby increasing the volume of TADs, without affecting the length of TADs"

It would be interesting to compare these volume effects to those recently reported by Hafner et al bioRxiv 2022 upon removing cohesin.

Despite the incongruencies of the TAD expands part, the interpration of the Hi-C largely parallels the recent chromosome tracing data :such as the "increased inter-TAD contact frequency" - inferred from the Hi-C and increased intra-TAD contact too.

14) The authors may wish to comment on the apparent incongruency between the Hi-C (increased contact at the TAD scale 0.3-6Mb) and the claim of "increased volume".

15) Fig 5 Labels:

-Figure 5B: the label on the plot is slightly confusing: I presume the normal samples are the grey boxes and the cancer samples are the colored boxes while the labels on x-axis reverse the order.

-Figure 5E: Is this RNA-seq in Hela cells overexpressing Rad21 or in the breast cancer cell lines?

16) Possible typo in conclusion in discussion:

"Therefore, we propose a model in which the total amount of cohesin (diffusive and chromatin bound) is a constant and up-regulated Rad21 leads to increased amount of Rad21-loader which promotes cohesin loading on the chromatin."

do the authors mean total amount of Rad21 is consant (if there's more loading of the cohesin complex, more of it must be on chromatin?) Hence the increase ratio of SMC3 in the 0.5M and 1M vs 0.1M fraction in Fig2B. No?

17) Similarly, 5-15 cohesin molecules were found in cohesin clusters in mES cells by live cell STED imaging(Gu et al., 2020). This was a little over-simplified.

From Gu et al: "the average distribution of the number of SMC3 molecules per cluster demonstrates that most clusters contain 2-5 molecules (Figure 3M)." [CTCF] coupled SMC3 clusters are enriched in clusters containing 5-15 molecules and depleted of clusters containing 1 or 2 molecules (Figure 3N, bottom)" Note, It is largely believed based on modeling (Fudenberg...Mirny 2016 Cell Reports), single cell chromosome tracing microscopy data (e.g. Bintu et al 2018 Science, Hafner 2022 Biorxiv) and live cell imaging (e.g. Gabriele...Hansen Science 2022) that most of chromatin-bound, loop-extruding cohesin is not residing anchored against CTCF sites.

**Authors’ response**

**Reviewer #1:**

Major points：

It would be useful to calculate cohesin's chromatin residence time from the iFRAP data shown in Figure 1F. It would also be interesting and important to compare the residence times of cohesin complexes in the absence and presence of RAD21 over-expression. Whereas WAPL depletion leads to vermicelli formation under conditions in which the residence time of cohesin on chromatin is greatly increased, this may be different after RAD21 over-expression, which one would not expect to lead to an increase in cohesin's residence time. If so, the implication would be that vermicelli can be formed simply by increasing the number of cohesin complexes on chromatin without an increase in cohesin's residence time. This would be an interesting finding.

**Author reply:**

Thanks for the reviewer’s valuable suggestions. To detect cohesin's residence time, we performed the FRAP assay in RAD21-EGFP mES cell line in the absence and presence of RAD21 over-expression (Figure R1). The photobleaching corrected intensity before and after photobleaching was normalized to 1 and 0. The FRAP curves were fitted by double exponential recovery,

Here and are the residence time and the faster one represents the non-topological binding of cohesin to chromatin while the slower one represents for topologically bound cohesin. Therefore, we used the slower one to estimate the residence time. The results showed that while the instant fluorescence recovery was low in bleached regions after RAD21 up-regulation, cohesin’s residence time on chromatin showed no difference.

As the reviewer mentioned that *the implication would be that vermicelli can be formed simply by increasing the number of cohesin complexes on chromatin without an increase in cohesin's residence time.* Our results were consistent with reviewer’s proposal, which indicated that vermicelli could be caused by accumulation of loaded cohesin, while cohesin’s unloading kinetics was not affected.

**Figure R1. (A)** Representative images of FRAP assay of SCC1(RAD21)-mAID-GFP ES cells in the absence or presence of RAD21 over-expression (scale bar is 5 μm). 3 independent experiments. Deconvolution was used to improve the imaging resolution and signal to noise ratio. **(B)** The fluorescence signals of unbleached regions were normalized to the first pre bleach image and plotted (mean ± S.D., n = 10 per condition). **(C)** Residence times for RAD21 in the absence or presence of RAD21 over-expression (n = 10 per condition; two sample T test; n.s., not significant)

2. Figure 1G and H, the authors find that the cohesin subunits RAD21, SMC1A and SMC3 are enriched in vermicelli, but that SA1 is not, and attribute this difference to the notion that the former three subunits form the cohesin ring, whereas SA1 is peripherally associated with this structure. However, SA1 has been found to be essential for loop extrusion (Davidson et al., 2019), implying that is should also be enriched in vermicelli if the cohesin complexes in these structures contain SA1, unless they contain the SA1 paralog SA2 instead. This possibility has not been considered by the authors. Maybe RAD21 over-expression favors the formation of cohesin complexes containing SA2, which in many cell types has been found to be 3-4 fold more abundant than SA1. It would therefore be interesting to compare SA1 and SA2 staining after RAD21 over-expression. It would also be better to use the standard nomenclature for SA1 and SA2, i.e. STAG1 and STAG2.

**Author reply:** Thanks for the reviewer’s valuable suggestions. To verify the colocalization of RAD21 and SA2, we used immunostaining assays in HeLa cells in the absence or presence of RAD21-OE. The results showed that the distribution of SA2 was co-localized with vermicelli, suggesting that formation of cohesin complexes contains SA2 (Figure R2 and Figure 1G&H). In line with the point that “in many cell types has been found to be 3-4 fold more abundant than SA1”, we found that the level of SA2 in the vermicelli is much higher than that of SA1.

Cohesin subunit SA1 is a protein that in human, which is encoded by the *STAG1* gene. Here we use SA1 and SA2 referring to the protein name of cohesin subunits, not the name of the gene, therefore SA1 and SA2 are more suitable.

**Figure R2.** Super-resolution images of cohesin subunits in the presence of RAD21 over-expression labelled with SA2 antibodies. Scale bar, 5 μm. Deconvolution was used to improve the imaging resolution and signal to noise ratio.

1) The authors speculate that RAD21 over-expression facilitates loading of cohesin onto DNA since over-expression of RAD21 mutants that are predicted to not interact with NIPBL does not cause vermicelli formation. However, there are several problems with this interpretation. First, the authors do not show that the RAD21 point mutants generated are unable to interact with NIPBL. This would be important to do.

**Author reply:** Thanks for the reviewer’s insightful suggestions. To detect whether RAD21 interacts with NIPBL, we tried co-immunoprecipitation and immunoblot assay against RAD21-GFP or NIPBL detect interaction between SMC3, NIPBL, WAPL and RAD21-GFP-WT/LIS-1-Ala/LIS-2-Ala. The results showed that two RAD21 mutants are unable to interact with NIPBL (Figure R3).

**Figure R3.** Co-immunoprecipitation and immunoblot assay against RAD21-GFP or NIPBL detect interaction between SMC3, WAPL, NIPBL with RAD21 using WCE obtained from HeLa cells with wild-type or LIS-Ala-mutant RAD21-OE.

2) Second, it is not clear if NIPBL functions as a loading factor for extruding cohesin complexes. The original proposal that NIPBL loads cohesin onto DNA was based on the notion that cohesin complexes which mediate sister chromatid cohesion, i.e. trans-contacts between replicated DNA molecules, entrap DNA inside cohesin's ring structure and on the observation that cells lacking NIPBL contain less cohesin on chromatin. The idea was that NIPBL promotes cohesin loading by transiently opening the cohesin ring so that DNA can be entrapped inside this structure to mediate cohesion. However, cohesin can perform loop extrusion, i.e form long-range DNA ciscontacts, without entrapping DNA inside its ring structure, and during loop extrusion NIPBL is continuously required, indicating that its role during this process might not be to load cohesin onto DNA (Davidson et al.,2019).

3) Third, cell lysates contain hetero-dimers of SMC1A and SMC3 (in early papers called '9S cohesin') which do not contain RAD21 (Losada et al., 1998; Sumara et al., 2000; Waizenegger et al., 2000), whereas '14S cohesin' on chromatin has a 1:1:1 stoichiometry of SMC1A, SMC3 and RAD21 (Holzmann et al., 2019). It is therefore well possible that RAD21 over-expression results in the assembly of cohesin complexes from the excess SMC1A-SMC3 hetero-dimers and that the resulting effective increase in cellular cohesin concentration would result in more cohesin complexes on chromatin and more chromatin looping events. The data presented in this manuscript can be equally well - if not better - explained by this alternative interpretation, since cohesin complexes containing overexpressed RAD21 mutants that cannot interact with NIPBL would also not extrude DNA and would therefore not accumulate in vermicelli. The manuscript should therefore discuss and consider both interpretations and needs to amend the conclusion that RAD21 over-expression promotes cohesin loading. RAD21 is presumably required for cohesin loading, but not any more than any of the other cohesin subunits.

**Author reply:** Thanks for reviewer’s constructive suggestions on the function of RAD21-NIPBL interaction.

To clarify these two alternative explanations, we performed a co-IP assay to detect cohesin formation. We found that the amount of cohesin remained stable after RAD21 up-regulation. Moreover, LC-MS quantification results showed that the copy number of soluble SMC3 did not exceed twice that of RAD21 in G2 and Prometaphase (Holzmann et al., 2019), so we do not think that RAD21 over-expression would lead to excess assembly of cohesin complexes using SMC1A-SMC3 heterodimer.

We transfected HeLa cells with plasmids containing HaloTag labelled cohesin subunits SMC1A, SMC3, RAD21, SA1 and SA2 with HaloTag, where only RAD21 over-expression led to overloading of cohesin onto chromatin, forming vermicelli structures. Therefore, we thought that RAD21 is the core subunit of the cohesin complex involved in directing genome organization.

Other points:

Line 87, 'cohesin' should be 'cohesion'.

**Author reply:** We thought ‘cohesin’ was reasonable here as most of the previous influential articles used this term (Haarhuis et al., 2017; Hansen et al., 2017; Rao et al., 2017).

Line 102 and 203-207, what is the evidence that in the imaging experiments single molecules were detected?

**Author reply:** The single molecule data were analyzed using the FIJI (Fiji Is Just ImageJ) plugin (Trackmate) software. The trajectory of individual H2A molecules is represented by lines of different colors (Figure 4R). Following Reviewer#2’s suggestion that since this result was not significantly related with the main findings, we have deleted the description about single-molecule tracking data from the text.

**Figure R4. (A)** The trajectory of individual H2A molecules is represented by lines of different colors. **(B)** Example histogram showing distribution of log_10_(diffusion coefficient) of single molecule motion and Gaussian fitting after tracking for 30000 frames (n>15 per condition).

Line 122-124, where the other cohesin subunits overexpressed at levels comparable to those of RAD21?

**Author reply:** We transfected HeLa cells with SMC1A, SMC3 and RAD21 at the same time and detected the distribution after 36 h. Given this prerequisite, we thought the levels of three subunits expression were comparable.

**Reviewer #2**

Major points：

Line 47-49 This claim does not seem to be proven by the data in the manuscript.

**Author reply:** We thank the reviewer for the constructive suggestions. We agreed that the experimental data do not directly support the loop extrusion model, and we have optimized the related description to avoid overstatement.

Line 146 The immunostaining of SA1 (Fig. 1G and Fig S1D) does not look convincing, there seems to be a problem with accessibility of the antibody, see e.g. (Schnell et al., 2012)

**Author reply:** We thank the reviewer for the suggestions. Since SA1 antibodies are rarely used for immunofluorescence, we selected an immunohistochemical SA1 antibody (abcam, ab4457) for immunofluorescence experiments. The results showed that the level of colocalization between SA1 and vermicelli was lower than the other cohesin subunits (Figure R1), and the statistic results of the colocalization was updated in Figure 1H.

**Figure R1.** Super-resolution images of cohesin subunits in the absence or presence of RAD21 over-expression labelled with SA1 antibody. Scale bar, 5 μm. Deconvolution was used to improve the imaging resolution and signal to noise ratio.

Scaling / display of fluorescence microscopy images should generally be improved. In some cases the images are so dark that the images cannot be assessed. Examples are: Fig. 1E, G/ 2G / 4G, H. Many images look deconvolved, please specify if deconvolution was used.

**Author reply:** Thanks for your suggestions. We have adjusted the scaling and improved the contrast of fluorescent images. All the images acquired by a spinning disk confocal system (Nikon, live SR CSU W) were deconvoluted to improve resolution and signal-to-noise ratio.

The authors use iFRAP to investigate whether cohesin is stably bound to chromatin or not. However, the data in Fig. 2 show that different pools of bound and unbound cohesin exist. Moreover, unbound RAD21 and RAD21 bound to NIPBL-MAU2 also exist in parallel (Fig. 6). These pools can be examined using standard FRAP. The experiment would require an analysis of the kinetics of the reappearance of fluorescence after a bleaching event. Of course, a full kinetic analysis of all mutants is beyond the scope of this manuscript, but the conclusions from Fig. 2 and the single molecule data (Figure S2E) could be substantially supported by classical FRAP experiments.

**Author reply:**

Thanks for the reviewer’s valuable suggestions. To detect cohesin's residence time, we performed the FRAP assay in RAD21-EGFP mES cell line in the absence and presence of RAD21 over-expression (Figure R2). The photobleaching corrected intensity before and after photobleaching was normalized to 1 and 0. The FRAP curves were fitted by double exponential recovery,

Here and are the residence time and the faster one represents the non-topological binding of cohesin to chromatin while the slower one represents for topologically bound cohesin. Therefore, we used the slower one to estimate the residence time. The results showed that while the instant fluorescence recovery was low in bleached regions after RAD21 up-regulation, cohesin’s residence time on chromatin showed no difference. Our results indicated that vermicelli could be caused by accumulation of loaded cohesin, while cohesin’s unloading kinetics was not affected.

**Figure R2. (A)** Representative images of FRAP assay of SCC1(RAD21)-mAID-GFP ES cells in the absence or presence of RAD21 over-expression (scale bar is 5 μm). 3 independent experiments. Deconvolution was used to improve the imaging resolution and signal to noise ratio. **(B)** The fluorescence signals of unbleached regions were normalized to the first pre bleach image and plotted (mean ± S.D., n = 10 per condition). **(C)** Residence times for RAD21 in the absence or presence of RAD21 over-expression (n = 10 per condition; two sample T test; n.s., not significant)

The two histograms in Figure S2E look identical, therefore the respective conclusions (line 205 - 206) cannot be assessed completely. However, from the statement in line 172 - 173 one had to expect a significant pool of RAD21 in cells overexpressing this protein. The authors should clarify this. In the methods section a description of the setup and the experimental conditions of the single molecule experiment are missing. In particular, it is unclear which cells were examined. Possibly only control cells were used. In this case the authors should consider to also measure cells overexpressing RAD21 which should result in an increase of the mobile fraction.

**Author reply:** We have replaced the correct Figure S2E in the revised version. Compared to the HeLa cell overexpressed H2A, we found that the majority of RAD21 molecules were stationary and only a small fraction was mobile after RAD21 overexpressed in HeLa cells, suggesting that up-regulated RAD21 was prone to bound stably to chromatin.

Following your suggestion that this result was not significantly related with the main findings, we have deleted the description about single-molecule tracking data from the text.

**Figure R3.** Example histogram showing distribution of log_10_(diffusion coefficient) of single molecule motion and Gaussian fitting after tracking for 30000 frames (n>15 per condition).

Figures 1H, 3B, 3C, 3F, 3I, S1B indicate significant differences, but the details of statistical tests performed on the data are not specified. The statistics in the other figures are outside of the expertise of the reviewer.

**Author reply：**Two sample t test was used in all figures mentioned above. Wilcoxon test was used in the sequencing data analysis as described in the previous publication (Chang et al., 2022).

Minor corrections

Line 203 I do not see how the described single molecule imaging helps to quantify the loading capacity. Please clarify this or delete this statement.

**Author reply:** We agree with the reviewer that the single-molecule data are not significantly related to major findings of our work, therefore we have removed the description about single-molecule tracking data from the text.

Line 64 - 65 The advantage of using STED microscopy is not obvious. The authors should clarify if really STED images are shown, as they look very similar in resolution to the others.

**Author reply:** Thanks for the reviewer’s valuable suggestions. We collected STED images using a Leica TCS SP8 STED microscopy system, and confocal images were collected by a live SR CSU W1 Nikon spinning disk confocal system with a super-resolution module. We have evaluated the spatial resolution using Image J plugin (Image Decorrelation Analysis). We found that the resolution of images acquired by live SR CSU W1 Nikon was about 0.189μm，while the resolution of STED images was about 0.077μm.

**Reviewer #3**

Major points：

1) RAD21 induces vermicelli-like chromosomes overlapping a vermicelli-like RAD21 beads-on-a-string configuration. This result was cool and striking. Three possible explanations are natural: that RAD21 is in limiting concentration, that exogenous RAD21 enhances loading, or that exogenous RAD21 interferes with unloading. The authors do a reasonable job in Figure 2 in arguing against the 1st explanation. From my reading I think it may still be possible that exogenous, tagged RAD21 interferes with unloading of cohesin. In the discussion the authors write: "In contrast, our findings showed that over-expression of cohesin subunit RAD21 led to accumulation of cohesin on chromatin and a vermicelli thread-like cohesin distribution in the nucleus of human HeLa cells, while cohesin release from chromatin mediated by Wapl remained unchanged. We therefore inferred that RAD21 is an important factor that affects cohesin loading independent of Wapl." .., but I don't believe that the authors showed any data supporting these conclusions about Wapl.

**Author reply:** We thank the reviewer for carefully evaluating our work and providing further constructive suggestions. As the reviewer thought there were not enough evidence to say that RAD21 affects cohesin loading independent of WAPL, we rephrased line #434-435 to avoid overstatements. In our work, we performed IP essay against WT/LIS1-Ala/LIS2-Ala RAD21-GFP and LIS1-Ala/LIS2-Ala did not affect RAD21-WAPL interaction, which indicates that the vermicelli pattern caused by excessive RAD21 might be independent of WAPL-mediated cohesin unloading. We also agree with the reviewer that more assays are still needed to explore the mechanism of RAD21 and WAPL in cohesin loading process in our further study.

1.1) If the authors bleach a region of the cell containing a fluorescently tagged RAD21, and measure recovery over time on timescales longer than currently estimated residence time of cohesin (~20 min an estimate probably good to within an order of magnitude), does it recover? Ideally this would be compared to the cohesin endogenously tagged with the same fluor (such lines exist, for example Hansen and colleagues engineered a Halo tagged cohesin in mESC, which could also be used for transfection of the over-expression construct. Making new endogenously tagged lines is non-trivial project, but if the expertise is available in house can be done in a few weeks to a month).

**Author reply:**

Thanks for the reviewer’s valuable suggestions. To detect cohesin's residence time, we performed the FRAP assay in RAD21-EGFP mES cell line in the absence and presence of RAD21 over-expression (Figure R1). The photobleaching corrected intensity before and after photobleaching was normalized to 1 and 0. The FRAP curves were fitted by double exponential recovery,

Here and are the residence time and the faster one represents the non-topological binding of cohesin to chromatin while the slower one represents for topologically bound cohesin. Therefore, we used the slower one to estimate the residence time. The results showed that while the instant fluorescence recovery was low in bleached regions after RAD21 up-regulation, cohesin’s residence time on chromatin showed no difference. Our results indicated that vermicelli could be caused by accumulation of loaded cohesin, while cohesin’s unloading kinetics was not affected.

**Figure R1. (A)** Representative images of FRAP assay of SCC1(RAD21)-mAID-GFP ES cells in the absence or presence of RAD21 over-expression (scale bar is 5 μm). 3 independent experiments. Deconvolution was used to improve the imaging resolution and signal to noise ratio. **(B)** The fluorescence signals of unbleached regions were normalized to the first pre bleach image and plotted (mean ± S.D., n = 10 per condition). **(C)** Residence times for RAD21 in the absence or presence of RAD21 over-expression (n = 10 per condition; two sample T test; n.s., not significant)

1.2) Does over-expression of an untagged RAD21 also create the vermicelli (in the DAPI channel)? This would rule out if the Halo-tag was impeding the unloading.

**Author reply:** We detected the distribution of chromatin by DNA staining using DAPI. The result showed vermicelli formation after untagged RAD21 over-expression, which rules out the possibility that the Halo-tag was impeding the unloading process (**Figure R2**).

**Figure R2.** Super-resolution images of HeLa cells in the absence or presence of untagged RAD21 over-expression. Scale bar, 5 μm. Deconvolution was used to improve the imaging resolution and signal to noise ratio.

(2) The differences in RAD21 organization in the cancer lines examined were very interesting. It might be beyond the scope of this work, but it would bolster the findings if by Hi-C these lines exhibited similar trends to enhanced near-cis and decreased far-cis interaction, as well as strengthened chromosome territories and weakened compartments. I'd be inclined to support publication even lacking these additional experiments, but I do think they'd enhance the impact of the paper and strengthen the claims here.

**Author reply:** We thank the reviewer for the constructive comments. This part of analysis aims to explore the pathological relevance of genome structural disorders caused by RAD21 up-regulation as illustrated in our manuscript, which may also shed light on the relationship between cancer and chromosomal proteins. So that we keep this part of results in the revised manuscript. We would like to thank the reviewer again for carefully evaluating this part of work.

3) It would have been nice to see a comparison or at least a discussion of RAD21 OE with the vermicelli chromosomes observed after WAPL degradation. For example, a more direct comparison of features reported in the literature due to removal of WAPL to the measures in this paper (including Hi-C features of TAD and loop sizes the minimum). If it is feasible in reasonable timeframe, experiments knocking down WAPL and comparison of RAD21 localization patterns (by IF or if the authors have and endogenously tagged RAD21 cell line) would be insightful.

**Author reply:** We thank the reviewer for the constructive suggestions about a more direct comparison of the features observed after RAD21 OE and WAPL depletion. We do agree with the reviewer’s comments and discuss the comparison more completely in the revised manuscript **line #407-423**, since we only compared features at the TAD scale in the initial submission.

To compare the Hi-C features caused by RAD21-OE and WAPL-depletion, we have done the Hi-C analyses referring to the methods of publications studying the WAPL-depletion (Haarhuis et al., 2017; Wutz et al., 2017). The results caused by RAD21-OE were similar with those after WAPL depletion. Specifically, we described them from macromolecular to micromolecular organization of chromosomes as follows.

1. In the genome-wide, the chromosomal contact frequency increased for short distance interactions and decreased for long distance interactions after RAD21-OE. The similar pattern was also found in WAPL-depleted cells reported by Haarhuis *et al.*
2. As for compartmentalization, over-expression of RAD21 resulted in less strict segregation between A and B compartments. Similarly, the compartments were also less clearly defined in WAPL-depleted cells (Haarhuis et al., 2017).
3. At the TAD scale, accumulated contacts were shown at TAD corners and inter-TAD interactions increased after RAD21 up-regulation, which were same as the feature resulted by WAPL depletion (Haarhuis et al., 2017). In addition, our work found that the TAD insulation score, TAD length and number remained unchanged after RAD21 over-expression. According to previous study, Wutz *et al* reported that TAD boundaries were weaken upon WAPL RNAi depletion, showing an increase in TAD size and a decrease in TAD number. While Haarhuis *et al* reported that WAPL depletion does not cause a reduction in TAD boundary strength. Wutz *et al* have also discussed the differences between their results were caused at least in part by differences in bioinformatics analyses (Haarhuis et al., 2017; Wutz et al., 2017). Based on the comparison and discussion on the results, we infer that the up-regulated RAD21 may cause TADs to be insulated stably and prevent TADs fusion, resulting in unchanged TAD insulation score, TAD length and number.

Given that we concentrated on the chromatin organization at the compartment and TAD scales, the resolution of Hi-C data in our work is insufficient to call loops. In addition, comparing with the macroscale chromatin contact alternatives in published studies (focusing on WAPL-depletion) could considerablly support our conclusions about RAD21-OE. So that the revised manuscript did not contain detailed analysis and discussion about chromatin loops, which is beyound the scope of this article but could be studied in our future work.

Minor concerns:

1) "High-resolution Hi-C data revealed that chromatin within some TADs is organized into small, stable contact domains anchored by CTCF/cohesin, known as loop domains (Rao et al., 61 2014; Rao et al., 2017)."

In the understanding of this reviewer, "contact domains" aren't really distinct from TADs in stability, size, or frequency of having corner loops. The Aiden lab preferred "contact domain" instead of "topologically associating domain", perhaps to avoid the suggestion that TADs have something to do with the mathematical idea of topology (knots).

**Author reply:** Thanks for reviewers’ comments on the introduction and defination of chromatin structure. We use the term “TADs” and “loop domains” to differentiate the chromatin structure at different scales. The former is about several kilobase while the later is usually hundreds of basepairs (Rao et al., 2014; Rao et al., 2017). We do agree that contact domains are not really distinct from TADs, and meanwhile we used the common terms just in order to illustrate that our Hi-C data analysis can compare the structure alternatives (RAD21-OE *vs.* control) at different scales.

2) "Wapl can drive the release of cohesin from chromatin (Ciosk et al., 2000; Gandhi et al., 2006; Kueng et al.,2006)… cells with Wapl deficiency(Haarhuis et al., 2017), suggesting that the formation of the vermicelli-like distribution is likely caused by excessive chromatin extrusion with the help of over-loaded cohesin. Inversely, PDS5 is necessary for the maintenance of cohesion (Panizza et al., 2000)."

I understood PDS5 to be an unloader like WAPL (e.g. see https://www.ncbi.nlm.nih.gov/pmc/articles/PMC6952610/), though the authors text left me with the opposite impression.

**Author reply:** We thank reviewers for the comments on the role of Pds5 in chromatin organization. According to the previous study, Pds5 has both positive and negative functions in cohesin regulation. On the one hand, Pds5 interacts with Sororin and stabilizes cohesin on chromosome and strengthens sister-chromatid cohesion. On the other hand, Pds5 engages and collaborates with Wapl to prompt the release of cohesin from chromosome (Ouyang et al., 2016). We have rephrased our statement in the revised manuscript in **line 85-86**.

4) it would be nice to see in Fig.1 the wild type (not RAD21 OE) chromosomes by DAPI and/or IF for RAD21. (this may already be in the supplement?). In Fig 1 and 2, It was unclear why the authors use iFRAP instead of FRAP, I thought the lack of recovery in the bleached region was much more convincing than the gradual photobleaching in the unbleached region. It seems to me a straight-forward quantification of the FRAP would be warranted here. I would also recommend a longer time course for the FRAP, given previous estimates that the loading/unloading kinetics of cohesin/RAD21 are much slower than for other transcription factors.

**Author reply:** Thanks for the reviewer’s insightful suggestions. The supplementary have showed super-resolution images of endogenous RAD21 and up-regulated RAD21 in nucleus (Figure S1A), as well as the measurement of RAD21 expression level by mean intensity quantification (Figure S1B).

Regarding the FRAP experiments, iFRAP was more suitable to detect the recovery of bleached region in LIS-mutant-RAD21, in which the fluorescent recovered too fast to be observed by FRAP. Following your suggestions, we also performed the FRAP assay in SCC1-mAID-GFP ESC cell line for a longer time course (more than 20 min) in the absence and presence of RAD21 over-expression. The results showed that while the instant fluorescence recovery was low in bleached regions after RAD21 up-regulation, cohesin’s residence time on chromatin showed no difference. Combining with co-IP results in Figure 2A, where the amount of cohesin remained stable, we proposed that vermicelli can be formed by increasing the amount of cohesin on chromatin, while cohesin’s unloading was not affected. Additional answer to this concern can be seen in major concern #1.1).

5) "We also got the pixel-based intensity distribution among different conditions to calculate their heterogeneity level (described in detail in the Methods section), which revealed that the distribution of RAD21 became more aggregated as its expression level increased (Figure 1D). "

Fig 1D is unclear in the plot and the main text. Either briefly explain in the text what the reader is supposed to take away from this panel or remove it. But it is currently unclear to me in what way this shows RAD21 being more aggregated. Panel C does make it look like the RAD21 vermicelli become more clearly resolved, but it is unclear from C whether this is just due to the increased levels and the saturation of the image pixels shown at 48 hrs.

**Author reply:** Thanks for the reviewer’s insightful suggestions. We defined the heterogeneity level to represent the aggregation degree of RAD21 (described in detail in the Methods section), and the fluorescent intensity was used to measure the expression level of RAD21. Figure 1D showed that with increasing time of RAD21 up-regulation in HeLa cells, the heterogeneity level was increased with the enhanced fluorescent intensity. This result suggested that the distribution of RAD21 became more aggregated as its expression level increased.

6) The clarity of the text could be improved for the general reader. Specifically:

- Line 183: the authors could be more specific in writing out 'loader', do they mean NIPBL?

**Author reply:** Thanks for your suggestions. ‘Loader’ in the text means NIPBL-MAU2 complex and we have rephrased our statement to avoid the confusion in the revised manuscript.

- Line 191: what do the authors mean by 'cohesin formation'. Does cohesin here refer to SMCs + SA + RAD21 complex?

**Author reply:** Yes, ‘cohesin formation’ means cohesin assembly from SMCs, RAD21 and SA.

- the authors define 'LIS' as RAD21-loader-interaction-site in line 187-188. It is a little confusing to then have LIS for SMC1a and SMC3. A sentence and or schematic of the position of the site within the protein in the text to introduce the knowledge about the loader interaction sites for each subunit would be helpful.

**Author reply:** ‘LIS’ means loader-interaction-site on the four cohesin subunits, not only for RAD21. The sequence was indentified by tiling peptide array to detect the loader interaction sites on the whole cohesin complex (Murayama and Uhlmann, 2014). We have modified the part accordingly in the revised manuscript.

-Fig. 2E - labeling Fig. 2E with not just LIS but RAD21-LIS-ala (as compared to 2G which is SMC1A or SMC3).

Adding wt RAD21 here as a comparison would be good, since this is GFP tagged and not Halo tagged. Also in 2F, wt as a comparison would be helpful to compare with what is expected.

**Author reply:** We collected FRAP data of RAD21-EGFP (wt RAD21), LIS1-ala, LIS2-ala, LIS1&LIS2-ala in the same batch of experiments. For the purpose of logical flow, we put wt RAD21-EGFP in Figure 1E while three mutants in Figure 2F.

7) Fig 2G SMC3 mutant -was unclear if this is an over-expression of the SMC3 loading mutant on top of the normal wildtype copies, or an edit of one or both of the endogenous copies. It would help the flow of the main text if the authors clarified in this in the main text directly - I don't think this sort of key information should be restricted to the supplemental methods.

**Author reply:** Thanks for reviewer’s suggestions. The SMC3 mutant is an over-expression of the SMC3 loading mutant on top of the normal wildtype copies. We have clarified this point in the revised manuscript in figure legend.

8) Fig. 3A/B: comparison for non-overexpressed RAD21 would be useful for how much CTCF-RAD21 colocalization happens in wt cells. -Fig. 3D: is control = RAD21 wt ?

**Author reply:** Thanks for the reviewer’s insightful suggestions. Privious studies have calculated the colocalization of CTCF and RAD21 (Anders S Hansen et al., eLife, 2017). In our work, we concerntrated more on the distribution of different proteins in the presence of RAD21 over-expression, and the colocalization of CTCF and RAD21 was shown in Figure 3A.

Fig. 3D: control means without RAD21-OE

9) "Bow-tying the TAD" (Fig 3E-G) This was interesting, though the bipartate nature of the structure in control condition as well as the RAD21 OE condition might reflect the cell cycle / paired sister chromatids – especially with the smallish n=20, differences in the fraction of G2 vs. G1 cells in each sample might contribute to difference in both volume and degree of separation (especially if RAD21 OE affects cell cycle / impairs M entry for example). This could be readily tested by adding a cell cycle marker like geminin into the quantification in parallel with the RAD21 label.

10) Does RAD21 OE effect cell cycle? (see also 6)

**Author reply:** Thanks for the reviewer’s insightful suggestions. We tried to analyze the cell cycle in the absence and presence of RAD21 over-expression by FACS (**Figure R3**). The results suggested that RAD21 over-expression has little effect on the cell cycle. Furthermore, vermicilli was also observed in the RAD21-OE cells after synchronization to G1/S phase in Figure S3C.

**Figure R3.** Cell cycle distribution of control and RAD21-GFP positive HeLa cells labeled with PI and analyzed by FACS (above). Gating strategy of FACS (blow).

11) I was unable to see these results due to the missing supplement: "1-hr EdU-Cy5 metabolic labeling (Li et al., 2021; Su et al., 2020) and found that the area of EdU clusters enlarged significantly after RAD21 was upregulated (Figures S3C and S3D)", how many clusters? What time point after metabolic labeling? Same cell cycle? Can enlarged EdU clusters be due to enhanced transcription?

**Author reply:** The missing supplement might cause some confusion. There are about 300-600 clusters in a cell. To incorporate EdU in HeLa cells, the cells were bathed with growing medium containing EdU for 30 min after releasing them from the G1/S transition for 1 h, followed by transfection and growth in fresh DMEM for 2 days, and all cells were in the same cell cycle.

By analysing RNA-seq data in our work, we calculated the TAD score (intra-TAD interaction/inter-TAD interaction) within differentially and stably expressed genes after RAD21 over-expression in HeLa cells, revealing that up-regulated genes had more significant reduced TAD score, in other words, up-regulated genes region has increased ratio of inter-TAD interaction. Based on these results, we speculate that enhanced transcription might be correlated with the chromatin structure alterations. Additionally, Boettiger *et al.* have studied the relationship between the median volumes and different chromatin state, and they found active domain (H3K4me2) was larger than repressed one (H3K27me3) with the same domain length (Boettiger et al., 2016). So that the enlarged EdU clusters are positively associated with enhanced transcription, but further evidence is needed to make a conclusion.

12) "median volume of TAD became larger in the presence of RAD21 up-regulation (Figure 3F), suggesting that decreased intra-TAD interactions resulted from excessive extrusion (Liu et al., 2021). These results under different conditions consistent with the CTCF/cohesin-anchored chromatin loop model" and "inside the TAD is more extended (larger volume shown in Figure 3G) due to the excessive extrusion of chromatin caused by overloaded cohesin, and therefore the interior structure of the chromatin region is more extended." - I suspect this is an artifact of the limiting labeling resolution (that more cohesin actually compacts the chromosome, not expanding it, but I see why the authors conclude the opposite. (Fig 3F, not G, no?) I think it's overstating things to say this expansion is consistent with the loop extrusion model - I think that's basically incorrect. I appreciate the cartoon intuition that extrusion creating a trajectory of the chromatin polymer away from the center - but this is not how it behaves in the chromatin polymer simulations cited nor how a floppy polymer is expected to behave in a crowded nuclear environment.

**Author reply:** Thanks for the reviewer’s insightful suggestions on our manuscript. We agree with the reviewer that the enlarged TAD may be resulted from the processive extrusion creating a trajectory of the chromatin polymer away from the center, instead of loop extrusion model or extention of the chromatin.

It is acceptable that up-regulation of cohesin actually compacts the chromatin resulting in vermicelli pattern rather than extending it. We have optimized description of the cartoon model in Figure 3G to avoid overstatement (line261 and line276 in the revised manuscript). We propose that the processive extrusion after RAD21-OE, where the chromatin is pulled away from the cohesin complex, results in a larger volume of TAD and chromosome territories.

13) "over-loaded cohesin can extend TADs when extruding progressively, thereby increasing the volume of TADs, without affecting the length of TADs" It would be interesting to compare these volume effects to those recently reported by Hafner et al bioRxiv 2022 upon removing cohesin.

Despite the incongruencies of the TAD expands part, the interpretation of the Hi-C largely parallels the recent chromosome tracing data: such as the "increased inter-TAD contact frequency" - inferred from the Hi-C and increased intra-TAD contact too.

**Author reply:** Thanks for the reviewer’s insightful suggestions. To address this concern, we have optimized related manuscript in **line 322-328,** adding the comments to compare the volume effects caused by removing cohesin.

In our study, we found that over-expressed RAD21 increased the volume of TADs. One recent imaging study on cohesin depleted cells reported that the contact distances increased significantly for all pairwise interactions both within and between TADs, resulting in TADs expansion (Hafner et al., 2022). Combining these results, we proposed that over-loaded cohesin can create a trajectory of the chromatin polymer away from the center when extruding progressively, thereby increasing the volume of TADs. While the cohesin loss might cause chromatin more disordered and increase the distance within and between TADs.­­

14) The authors may wish to comment on the apparent incongruency between the Hi-C (increased contact at the TAD scale 0.3-6Mb) and the claim of "increased volume".

**Author reply:** Thanks for the reviewer’s insightful suggestions. These two conclusions were produced by Hi-C data analyses and FISH separately, and we thought the incongruency can be explained as we showed in cartoon Figure 3G. On the one hand, the over-loaded cohesin promoted the progressive extrusion of chromatin and resulted in a trajectory of the chromatin polymer away from the center, which displayed a larger volume of TAD. Simultaneously, progressive extrusion of chromatin could increase the contact frequency of adjacent chromatin (maybe increased inter-TAD interaction), so that Hi-C experiment captures more contact basepairs at short-distance interaction (0.3-6Mb).

15) Fig 5 Labels:

-Figure 5B: the label on the plot is slightly confusing: I presume the normal samples are the grey boxes and the cancer samples are the colored boxes while the labels on x-axis reverse the order.

-Figure 5E: Is this RNA-seq in Hela cells overexpressing RAD21 or in the breast cancer cell lines?

**Author reply:** We reversed the order of the boxes in Figure 5B, which can be found in the revised manuscript.

RNA-seq was performed using Hela cells over-expressing RAD21.

16) Possible typo in conclusion in discussion: "Therefore, we propose a model in which the total amount of cohesin (diffusive and chromatin bound) is a constant and up-regulated RAD21 leads to increased amount of RAD21-loader which promotes cohesin loading on the chromatin."

Do the authors mean total amount of RAD21 is constant (if there's more loading of the cohesin complex, more of it must be on chromatin?) Hence the increase ratio of SMC3 in the 0.5M and 1M vs 0.1M fraction in Fig 2B. No?

**Author reply:** Based on our results, we found that up-regulation of RAD21 increased the amount of RAD21 by 2.5-fold while the total amount of cohesin showed almost no difference (Fig 2A, we have reorganized Figure2 A-C). Therefore we proposed the model that up-regulated RAD21 facilitates RAD21-loader interaction and promotes cohesin loading on the chromatin, while the total amount of cohesin remained unchanged.

17) Similarly, 5-15 cohesin molecules were found in cohesin clusters in mES cells by live cell STED imaging (Gu et al., 2020). This was a little over-simplified.

From Gu et al: "the average distribution of the number of SMC3 molecules per cluster demonstrates that most clusters contain 2-5 molecules (Figure 3M)." [CTCF] coupled SMC3 clusters are enriched in clusters containing 5-15 molecules and depleted of clusters containing 1 or 2 molecules (Figure 3N, bottom)" Note, It is largely believed based on modeling (Fudenberg...Mirny 2016 Cell Reports), single cell chromosome tracing microscopy data (e.g. Bintu et al 2018 Science, Hafner 2022 Biorxiv) and live cell imaging (e.g. Gabriele...Hansen Science 2022) that most of chromatin-bound, loop-extruding cohesin is not residing anchored against CTCF sites.

**Author reply:** We thank the reviewer for the constructive suggestions. We have revised the manuscript as follows.

Similarly, 2-5 molecules in average were found in cohesin clusters in mES cells by live cell STED imaging while CTCF coupled cohesin clusters contain 5-15 molecules(Gu et al., 2020).

**Reference**

Boettiger, A.N., Bintu, B., Moffitt, J.R., Wang, S., Beliveau, B.J., Fudenberg, G., Imakaev, M., Mirny, L.A., Wu, C.T., and Zhuang, X. (2016). Super-resolution imaging reveals distinct chromatin folding for different epigenetic states. Nature *529*, 418-422.

Chang, L., Li, M., Shao, S., Li, C., Ai, S., Xue, B., Hou, Y., Zhang, Y., Li, R., Fan, X.*, et al.* (2022). Nuclear peripheral chromatin-lamin B1 interaction is required for global integrity of chromatin architecture and dynamics in human cells. Protein Cell *13*, 258-280.

Gu, B., Comerci, C.J., McCarthy, D.G., Saurabh, S., Moerner, W.E., and Wysocka, J. (2020). Opposing Effects of Cohesin and Transcription on CTCF Organization Revealed by Super-resolution Imaging. Mol Cell *80*, 699-711 e697.

Haarhuis, J.H.I., van der Weide, R.H., Blomen, V.A., Yanez-Cuna, J.O., Amendola, M., van Ruiten, M.S., Krijger, P.H.L., Teunissen, H., Medema, R.H., van Steensel, B.*, et al.* (2017). The Cohesin Release Factor WAPL Restricts Chromatin Loop Extension. Cell *169*, 693-707.

Hansen, A.S., Pustova, I., Cattoglio, C., Tjian, R., and Darzacq, X. (2017). CTCF and cohesin regulate chromatin loop stability with distinct dynamics. Elife *6*.

Holzmann, J., Politi, A.Z., Nagasaka, K., Hantsche-Grininger, M., Walther, N., Koch, B., Fuchs, J., Durnberger, G., Tang, W., Ladurner, R.*, et al.* (2019). Absolute quantification of cohesin, CTCF and their regulators in human cells. Elife *8*.

Murayama, Y., and Uhlmann, F. (2014). Biochemical reconstitution of topological DNA binding by the cohesin ring. Nature *505*, 367-371.

Ouyang, Z., Zheng, G., Tomchick, D.R., Luo, X., and Yu, H. (2016). Structural Basis and IP6 Requirement for Pds5-Dependent Cohesin Dynamics. Mol Cell *62*, 248-259.

Rao, S.S., Huntley, M.H., Durand, N.C., Stamenova, E.K., Bochkov, I.D., Robinson, J.T., Sanborn, A.L., Machol, I., Omer, A.D., Lander, E.S.*, et al.* (2014). A 3D map of the human genome at kilobase resolution reveals principles of chromatin looping. Cell *159*, 1665-1680.

Rao, S.S.P., Huang, S.C., Glenn St Hilaire, B., Engreitz, J.M., Perez, E.M., Kieffer-Kwon, K.R., Sanborn, A.L., Johnstone, S.E., Bascom, G.D., Bochkov, I.D.*, et al.* (2017). Cohesin Loss Eliminates All Loop Domains. Cell *171*, 305-320 e324.

Wutz, G., Varnai, C., Nagasaka, K., Cisneros, D.A., Stocsits, R.R., Tang, W., Schoenfelder, S., Jessberger, G., Muhar, M., Hossain, M.J.*, et al.* (2017). Topologically associating domains and chromatin loops depend on cohesin and are regulated by CTCF, WAPL, and PDS5 proteins. EMBO J *36*, 3573-3599.

**2^nd^ round**

**Reviewer 1**

In the revised version of their manuscript, the authors have addressed some but not all of my comments and concerns. As before, I find that the observation that RAD21 overexpression causes vermicelli interesting and worthy of publication, but I am not convinced at all by the authors’ proposal that this phenomenon reflects a role of RAD21 in cohesin loading.

Major points

1. The authors propose that RAD21 overexpression facilitates loading of cohesin onto DNA and make very strong claims about this throughout their manuscript, but do not provide sufficient evidence for this hypothesis and not even an explanation of why this could be the case. RAD21 is essential for chromatin binding of cohesin and accordingly subunit stoichiometry measurements have indicated that all cohesin complexes on chromatin contain RAD21 (Holzmann et al., 2019). If so, how could overexpression of RAD21 facilitate the loading of cohesin complexes that already contained RAD21 to start with? If anything, the opposite effect would be expected, namely that overexpressed RAD21 should compete with cohesin for NIPBL binding and thus the amount of cohesin on chromatin should be reduced, not increased. The authors need to provide at least a hypothetical model for how overexpressed RAD21 could facilitate cohesin loading, both in the text of their manuscript and in Figure 6. As it stands, the model shown in this Figure does not provide an explanation.

2. As pointed out in my previous review, there is an alternative explanation for the authors’ observation that is much more plausible. In contrast to cohesin subunits on chromatin, cohesin’s ring forming subunits do not exist in stoichiometric ratios in soluble fraction. In these, SMC1 and SMC3 exist in considerable excess over RAD21. This can be detected as the presence of SMC1-SMC3 heterodimers in a 9S fraction by density gradient centrifugation that is distinct from “14S cohesin” which contains in addition RAD21 and STAG subunits (Losada et al., 2000; Sumara et al., 2000), and also by quantitative mass spectrometry (Holzmann et al., 2019). This implies that RAD21 levels are limiting for cohesin assembly and thus also for the amounts of cohesin that can associate with chromatin (for which - as said – RAD21 is essential). The situation in vertebrate cells is therefore similar to the one in budding yeast, where Scc1 (the yeast ortholog of RAD21) is rate limiting for cohesin assembly and where cohesin can therefore only associate with chromatin once Scc1 has been synthesized at the end of G1-phase (Michaelis et al., 1997). I cannot see any clear evidence in the revised manuscript that would contradict this simpler and thus more plausible explanation. The finding that RAD21 mutants, which cannot interact with NIPBL, do not increase cohesin association also fits perfectly well with this alternative explanation, since assembly of this RAD21 mutant into cohesin would result in cohesin complexes that cannot associate with chromatin. The authors need to point out this alternative interpretation more clearly. If they wanted to reject it, they would need stronger experimental support and at least a hypothetical model for how RAD21 overexpression could facilitate cohesin loading without assembly of new cohesin complexes. If such data and hypotheses cannot be generated, the authors should consider this alternative explanation much more seriously and not just reject it.

3. The authors report that only little STAG1 can be detected in vermicelli upon overexpression of RAD21. They explain this by the notion that STAG1 is not part of the cohesin ring. However, STAG2 – which can easily be detected in vermicelli – is also not part of the cohesin ring. This explanation is therefore not logical. Furthermore, the authors now show that the levels of STAG1 are much below those of STAG2, implying that the lack of detectability of STAG1 in vermicelli could simply be due to its lower abundance. The authors should mention this possibility.

Other points (in order in which they appear in the manuscript)

I had mentioned some of these comments before but am listing them here again, now adjusted to the line numbers in the revised manuscript, because the authors ignored them and did not provide an explanation for why they did not consider them. I have to say this is a bit irritating because reviewing a manuscript is labor intensive and a service to the authors.

Line 66, Gassler et al., 2017; Wutz et al., 2017 should be added.

Line 78, Ciosk et al., 2000 did not report findings related to Wapl.

Line 83, Wutz et al., 2017 should be added.

Line 88, ‘loading complex’ should be ‘NIPBL’ since domains of this protein and not of the loading complex are being described.

Line 133/136 It is misleading to use the term “aggregation” to describe the accumulation of RAD21 in vermicelli since this implies non-specific interactions between proteins, as seen in protein aggregates caused by misfolding. The term “accumulation” would be better.

Line 325 The Hafner et al., 2022 reference is missing in the reference list.

**Reviewer 2**

Dear Authors,

Thank you very much for your work, I have read the substantially revised version with great pleasure. Especially the section "RAD21-loader Interaction Facilitates Vermicelli-like Structure Formation by Promoting Cohesin Loading" has a much better readability. You have implemented my suggestion regarding the kinetic analyses of the FRAP data excellently. The kinetic analysis of the FRAP data (Figure S1E and S1F) clearly shows that up-regulation of RAD21 expression does not change the cohesin residence time.

I only noticed some small inaccuracies:

Sometimes there are spaces between the text and the literature citation and sometimes it is missing (e.g. line 53 and 56).

Figure S1B is not mentioned in the text

Line 118 / 119 Halo Tag is mentioned two times

Line 227 Grammar

Line 264 Space is missing

Line 274 Figure 3F not 3E

Line 325 (Hafner et al., 2022) is missing in the bibliography

**Reviewer 3**

The authors have addressed my concerns and I support speedy publication.

Further comments from Reviewer 3 regarding Reviewer 1’s comments:

I concur with Reviewer 1 -- I find the rate-limiting RAD21 for assembly model a more plausible explanation of these interesting findings.

However, to my reading of the text, I didn't share Reviewer 1's impression that the authors were conclusively rejecting the rate-limiting model (model 2 above) and advocating for a RAD21 is an enzymatic loader of cohesin complexes that already contain a structural copy of RAD21 (model 1). When the authors conclude that RAD21 overexpression facilitates more cohesin loading, I did not read this to mean they were suggesting RAD21 substitutes for NIBPL and loads more cohesin on -- though I can see how a very literal reading of Fig 6 might suggest this.

I do think the manuscript would be strengthened if the text (and possibly the model cartoon) were tweaked to clarify what is being claimed and what is ruled out. If in the discussion the authors wish to indicate they favor a novel loading model, distinct from what is believed to occur in yeast, I'm fine with such speculation, though it is I'd say it is a hypothesis permitted or inspired by the data, which does not yet rule out the (simpler) explanation of rate-limiting-RAD21 levels.

**Authors’ responses**

**Reviewer #1**

Major points：

1. The authors propose that RAD21 overexpression facilitates loading of cohesin onto DNA and make very strong claims about this throughout their manuscript, but do not provide sufficient evidence for this hypothesis and not even an explanation of why this could be the case. RAD21 is essential for chromatin binding of cohesin and accordingly subunit stoichiometry measurements have indicated that all cohesin complexes on chromatin contain RAD21 (Holzmann et al., 2019). If so, how could overexpression of RAD21 facilitate the loading of cohesin complexes that already contained RAD21 to start with? If anything, the opposite effect would be expected, namely that overexpressed RAD21 should compete with cohesin for NIPBL binding and thus the amount of cohesin on chromatin should be reduced, not increased. The authors need to provide at least a hypothetical model for how overexpressed RAD21 could facilitate cohesin loading, both in the text of their manuscript and in Figure 6. As it stands, the model shown in this Figure does not provide an explanation.

2. As pointed out in my previous review, there is an alternative explanation for the authors’ observation that is much more plausible. In contrast to cohesin subunits on chromatin, cohesin’s ring forming subunits do not exist in stoichiometric ratios in soluble fraction. In these, SMC1 and SMC3 exist in considerable excess over RAD21. This can be detected as the presence of SMC1-SMC3 heterodimers in a 9S fraction by density gradient centrifugation that is distinct from “14S cohesin” which contains in addition RAD21 and STAG subunits (Losada et al., 2000; Sumara et al., 2000), and also by quantitative mass spectrometry (Holzmann et al., 2019). This implies that RAD21 levels are limiting for cohesin assembly and thus also for the amounts of cohesin that can associate with chromatin (for which - as said – RAD21 is essential). The situation in vertebrate cells is therefore similar to the one in budding yeast, where Scc1 (the yeast ortholog of RAD21) is rate limiting for cohesin assembly and where cohesin can therefore only associate with chromatin once Scc1 has been synthesized at the end of G1-phase (Michaelis et al., 1997). I cannot see any clear evidence in the revised manuscript that would contradict this simpler and thus more plausible explanation. The finding that RAD21 mutants, which cannot interact with NIPBL, do not increase cohesin association also fits perfectly well with this alternative explanation, since assembly of this RAD21 mutant into cohesin would result in cohesin complexes that cannot associate with chromatin. The authors need to point out this alternative interpretation more clearly. If they wanted to reject it, they would need stronger experimental support and at least a hypothetical model for how RAD21 overexpression could facilitate cohesin loading without assembly of new cohesin complexes. If such data and hypotheses cannot be generated, the authors should consider this alternative explanation much more seriously and not just reject it.

Author reply to Q1&2：

We thank the reviewer for the constructive suggestions. In the current work, we discovered the formation of vermicelli after overexpressing RAD21 in wild-type HeLa cells. Accordingly, biochemical assay confirmed that the proportion of cohesin bound to chromatin was significantly increased while the proportion of free cohesin was significantly reduced after vermicelli formation (Figure 2B&2C). Interestingly, in the cell over-expressing RAD21, while the level of RAD21 was increased about 2 folds, the total amount of cohesin (whole cell extract by immunoprecipitation against SMC3) showed almost no changes (Figure 2A).

Given that SMC1 and SMC3 exist in considerable excess over RAD21 (Losada et al., 2000; Sumara et al., 2000; Holzmann et al., 2019), this suggests that RAD21 may be the limiting factor for the amount of cohesin. Therefore, over-expressing RAD21 would lead to more cohesin as well as loader-bound cohesin, which increases the level of cohesin loaded on chromatin, consistent with our observation in Figure 2B&2C. Considering the level of RAD21 was only increased by 2 folds in RAD21-OE cells, there would not be competition in binding loader between RAD21 and cohesin. Regarding the observation that the total amount of cohesin was not increased as much as RAD21 in RAD21-OE cells, this might be attributed to the crucial role of cohesin in organizing chromatin structure, which requires strict regulation to maintain the homeostasis of cohesin levels in a way to degrade cohesin unassociated with chromatin. Based on these data and analysis, we agree with the reviewer and restate our model as the following:

Up-regulated RAD21 promotes the formation of both RAD21-loader and cohesin, and in turn increases the number of cohesin-loader complexes and cohesin loading on chromatin. Then, the over-loaded cohesin excessively extrudes the chromatin into a vermicelli-like morphology with RAD21 clustered into “beads” and bow-tying a TAD to form a “beads on a string” pattern.

3. The authors report that only little STAG1 can be detected in vermicelli upon overexpression of RAD21. They explain this by the notion that STAG1 is not part of the cohesin ring. However, STAG2 – which can easily be detected in vermicelli – is also not part of the cohesin ring. This explanation is therefore not logical. Furthermore, the authors now show that the levels of STAG1 are much below those of STAG2, implying that the lack of detectability of STAG1 in vermicelli could simply be due to its lower abundance. The authors should mention this possibility.

Author reply：

Thanks for the reviewer’s valuable suggestions.

Our immuno-stained results (Figure 1G and 1H) showed that SA1 was less enriched in the vermicelli-like structures in comparison with SA2, we therefore proposed that SA2 may be more responsible for arresting cohesin in that RAD21 mediated vermicelli pattern. As the reviewer mentioned that the difference of colocalization level could also due to lower abundance of SA1, we do agree with this possible explanation, and we optimized our statement in the revised manuscript. We suggested that the less enrichment of SA1 in the vermicelli-like structures could be due to its lower abundance in the cell.
